# Supplementary material for: Transport of impact ejecta from Mars to its moons as a means to reveal Martian history
Source: Sci Rep. 2019 Dec 27;9:19833. doi: 10.1038/s41598-019-56139-x (PMC6934779; doi:10.1038/s41598-019-56139-x)
Supplement: Supplementary file 1 — Supplementary Information [file 41598_2019_56139_MOESM1_ESM.pdf]

Supplementary Materials for

Transport of impact ejecta from Mars to its moons as a means to reveal Martian history

*Ryuki Hyodo, Kosuke Kurosawa, Hidenori Genda, Tomohiro Usui, Kazuhisa Fujita,

Correspondence to: hyodo.ryuki@jaxa.jp

This PDF file includes:

Materials and Methods
Supplementary Text
Figs. S1 to S20
Tables S1

Materials and Methods

SPH simulations for crater-forming impacts

The mass and velocity distributions of Martian materials ejected by impacts are key to more precisely estimating the mass accreting on Martian moons. Here, we used the three-dimensional smooth particles hydrodynamic (SPH) method[32,33] to perform impact simulations. The SPH method can easily trace large deformations and shock waves, which are involved in planetary impact phenomena. Our numerical code was the same as that used in Kurosawa et al. (2018)[34]. It can calculate a purely hydrodynamic flow without gravity and material strength.

We numerically calculated an impact of a spherical projectile with a radius of $R_p = 10$ km onto the flat surface of a half-sphere target with a radius of five times the projectile radius. For the numerical resolution, 5.2×10^5 SPH particles were used for the projectile, which corresponds to about 50 SPH particles per projectile radius (50PPPR). The equal-mass SPH particles were used for the target, which corresponds to 3.3×10^7 SPH particles for the target. The Tillotson equation of state[35] with the parameter sets for granite[36] was used for both the projectile and target. For the impact conditions, we considered various impact velocities from 6 km/s to 18 km/s with a 3 km/s interval and various impact angles from 0 degrees (vertical impact) to 75 degrees with a 15-degree interval. In total, 30 impact simulations were performed. Although just one set was considered for the impactor radius, we are able to convert our results to any size of impactor because all hydrodynamic equations can be rewritten in a dimensionless form in cases without gravity and strength[37].

Figure S1 provides snapshots of the head-on and 45-degree oblique impacts at the impact velocity of 12 km/s. The head-on impact develops a symmetric pattern of ejecta, whereas the oblique impact produces an asymmetric one in which downstream ejecta dominates. Clearly, high-speed ejecta is produced more efficiently by the 45-degree impact than the head-on impact. Figure S2 shows the peak pressure experienced by ejected materials. The amount of high-speed ejecta that could potentially reach Phobos and experience relatively low peak pressure (e.g., < 10 GPa) is much greater for the 45-degree impact than for the head-on impact.

Figure S3 shows the ejected mass of the target materials (i.e., Martian materials) whose ejection velocity exceeds 3.8 km/s for all impact simulations with various impact velocities and angles. Note that a velocity greater than 3.8 km/s is needed for ejected materials to reach the current orbit of Phobos. As mentioned in the following section, the radii of Phobos and Deimos were expressed as being 50 times larger to save computation costs for estimating the collision probability of these ejected materials on Phobos or Deimos. Since the moon has a physical size, some particles can reach Phobos with smaller ejection velocity than 3.8 km/s. In order to consider the effect of the physical radius of the moon, we use ejecta particles whose velocity is larger than 3.5 km/s.

Figure S3 also shows that the ejected mass normalized by the impactor mass (M_{imp}) increases with the impact velocity and has a peak at the impact angle of 45 degrees. At the same impact velocity, the ejected mass for a 45-degree impact is more than a factor of 3 greater than that for a 90-degree vertical impact. It is also important to note that to produce a crater of the same size at the same impact velocity (see Eq. (S1)), the impactor size must be increased by 1.15 times (or the impactor mass must be increased by 1.15^3–1.5 times) at the 45-degree impact compared to

the 90-degree impact. Therefore, the ejection mass for a crater of the same size is increased by more than $3 \times 1.5 = 4.5$ times for a 45-degree oblique impact compared to 90-degree vertical one at the same impact velocity.

We stored the data of velocity and position relative to the impact point for all SPH particles having a particle velocity greater than 3.5 km/s, and we used those data as the initial condition of the orbital calculations of ejecta from impact point to Martian moons.

In impact calculations, lightly shocked ejecta comes from near the surface of the target; ejecta excavated deeper from the surface experienced heavier shock. SPH calculations may have difficulty properly resolving the shock levels of near-surface material[34]. Therefore, to check the resolution effects of our 3D SPH calculations, we perform calculations for the high- and low-resolution cases (100 PPPR and 25 PPPR, respectively) in addition to our nominal case of 50 PPRP where $v_{imp} = 12$ km/s with impact angles of $\theta = 30$ and 45 degrees. These parameters are reasonable considering that the mean impact parameters used in this work are $v_{imp} = 14$ km/s with $\theta = 45$ degrees. Figure S4 shows the lowest peak pressures of the 2nd, 3rd and 4th upper layers from the surface layer (i.e., the 1st layer) of the target (Mars) whose ejection velocities are 3.8 km/s. The resolution effects of our 3D SPH code have been investigated previously[34] and show that the relationship between peak shock pressures and ejection velocities for particles initially located at layers deeper than the 3rd layer from the free surface is reliable. As seen at infinite spatial resolutions ($1/N_{PPPR} = 0$; Figure S4) obtained from the fitted lines of the three different resolution cases (25 PPRP, 50 PPRP, and 100 PPRP) for the different layers, the lowest peak pressures for the case of 45 degrees exceed 5 GPa for all the layers studied here. However, the lowest peak pressures at the infinite spatial resolutions for the case of 30 degrees shows $P_{peak} < 5$ GPa. Thus, the Mars ejecta delivered to Phobos includes less shocked (< 5 GPa) and more fragile materials than those of the Martian meteorites (> 5 GPa) such that one part of our main results is not qualitatively affected by the resolution of the SPH simulations.

Impact conditions

We considered five fresh craters on Mars as sources of Martian rocks on the moons. Their locations and diameters are summarized in Table S1. We searched the set of the impact conditions, characterized by projectile diameter D_p, impact velocity v_{imp}, and impact angle θ measured from the horizontal, using a Monte Carlo approach to investigate the effects of the stochastic nature of impact events. The size distribution of the impactor was taken from the crater size–frequency distribution[18]. We assumed that the impact velocity distribution can be approximated as a Rayleigh distribution[38]. The mean impact velocity onto Mars was estimated to be 14 km/s by assuming the dynamical transports of the impactors from $\boldsymbol{\nu}_6$ mean motion resonance in the main-belt region[39]. The impact angle distribution was taken from $\sin(2\theta)$[40]. To combine them with the impact conditions employed in the SPH simulation, we obtained the impact velocities and angles discretely from the original distributions. The ranges of impact velocity and angle were set to 6 km/s to 18 km/s at a step of 3 km/s and 15 degrees to 90 degrees at a step of 15 degrees, respectively.

The transient crater diameter D_{tr} under a gravity-dominated regime was calculated by the π-group scaling laws[41] as follows:

$$D_{tr} = \left(\frac{\pi}{6}\right)^{\frac{1}{3}} C_D \left(\frac{4\pi}{3}\right)^{-\frac{\beta}{3}} \left(\frac{\rho_p}{\rho_t}\right)^{\frac{1}{3}} D_p^{1-\beta} g^{-\beta} \left(v_{imp} \sin\theta\right)^{2\beta}, \quad (S1)$$

where $C_D = 1.4$, $\beta = 0.17$, $\rho_p = 2.7$ Mg/m^3, $\rho_t = 2.7$ Mg/m^3, and $g = 3.7$ m/s^2 are a dimensionless scaling constant, a dimensionless scaling exponent, projectile density, target density, and gravitational acceleration, respectively. The scaling parameters correspond to the values for quartz sand[41]. We assumed that the projectile and the Martian crust are basaltic rock. An empirical equation was employed to estimate the final crater diameter D_f from the transient diameter:

$$D_f = 1.2 D_c^{-0.13} D_{tr}^{1.13}, \quad (S2)$$

where D_c is the transition diameter from simple craters to complex craters[42]. The transition diameter D_c on Mars was estimated to be 7 km based on remote sensing data[43]. We extracted 10,000 events for each crater on Mars, which reproduced the observed diameters to within ±30 m, from the Monte Carlo calculations. Figure S5 shows D_f as a function of D_p. The dispersion in D_p comes from the distribution of the normal component of impact velocity (see Eq. (S1)). Hereafter, we use the averaged values of the projectile diameters. The standard deviations in the projectile diameters are ~20%. The calculations described above provide the absolute values of the total mass of high-speed ejecta that reaches Martian moons in combination with a hydrocode calculation. Figure S6 shows the high-speed ejecta masses at velocities higher than 3.8 km/s calculated by the 3-D SPH computations as a function of the final crater diameters estimated by Eqs. (S1) and (S2). The velocity threshold, 3.8 km/s, corresponds to the minimum launch speed required to reach the orbit of Phobos. In the case of transport to Deimos, the value is 4.5 km/s. The high-speed ejecta mass depends largely on the impact angle. Moderately oblique impacts (30–60 degrees from the tangent plane) efficiently produce high-speed ejecta[44]. We also show the total excavated mass as the black line estimated by a combination between D_{tr} and the Maxwell's Z model[45,46] with $Z = 3$, showing that the total mass of the high-speed ejecta is 1–2 orders of magnitude smaller than the total excavated mass. Note that the high-speed ejecta mass does not depend strongly on the impact velocity, although the point size in the figure corresponds to the impact velocities from 6 to 18 km/s (small to large).

Two-body orbital calculation

In this work, we randomly picked up combinations of the large crater-forming impact parameters (v_{imp}, m_{imp}, and θ_{imp}) to form the crater and orbital phase of the Martian moon (the orbital plane was assumed to be on the equatorial plane of Mars). In the case of the five large craters, we fixed the crater-forming impact locations at the current crater locations. The impact direction of the craters on Mars was chosen randomly, except for the case of Zunil. Because the Zunil Crater is the largest known ray crater, with a diameter larger than 10 km[47], the geology of Zunil and the area around the crater, including the spatial distribution of the ray system and the secondary craters, has been studied extensively[15,47,48,49]. The impact forming Zunil Crater was constrained to a moderately oblique impact from the east–northeast[49]. Thus, the impact direction for Zunil Crater in our model was chosen randomly from northeast to east. Also, there is another important parameter, the phase angle of the moons at the time of the impacts. The phase angles were also randomly chosen from within 360 degrees. In the fully randomized case, the latitude

and longitude of the impact were chosen randomly. At each of the five large craters (Mojave, Tooting, Corinto, McMurdo, and Zunil) and in the fully randomized case, we performed 10,000 and 30,000 times Monte Carlo simulations, respectively.

Using the selected crater-forming impact parameters, we found the outcomes of the SPH simulations to obtain positions and velocities of the ejecta. Then, we passed this information to the orbital calculation as inputs.

In the orbital calculation, we assumed that the ejecta and the moons are massless point particles because they are significantly small compared to Mars. Phobos and Deimos were assumed to have a circular orbit with the current semi-major axis. A single orbit of the ejecta and the moons was solved analytically by considering a two-body problem with Mars. In the two-body problem, we could solve the location and velocity at an arbitrary time if we knew the initial position and velocity of the ejecta[50]. To increase the resolution of the impact detection, we inflated the diameter of the moons by 50 times (inflation factor $f = 50$) by following the method employed in a previous study[20], but we confirmed that the results do not change significantly when $f < 100$ (the results of the impact number are finally scaled by f^2).

We detected a collision when the relative distance between a Martian moon and ejecta was smaller than the radius of the moon. We confirmed that the outcome obtained from our two-body analytical approach is consistent with that obtained from a direct *N*-body calculation where orbits of test particles are integrated numerically under the gravity of Mars [the same *N*-body code is used in Hyodo et al., (2015)[51] and Hyodo & Charnoz, (2017)[52]]. Because *N*-body calculation is time-consuming, we used the above analytical approach in this work.

Supplementary Text

S1. Basic physics of the impact debris

S1.1. Orbits of the impact debris

Crater-forming impacts produce ejecta from near the surface of Mars. Because the impact debris rarely has a velocity vector parallel to the surface of Mars and because a Keplerian orbit is a closed orbit (when eccentricity $e < 1$), the impact debris with velocity smaller than the escape velocity of Mars (less than ~5 km/s) intrinsically has a pericenter distance $a_{\rm peri}$ smaller than the radius of the impacted planet $R_{\rm Mars}$ (Mars radius), and the semi-major axis a and eccentricity e of the debris satisfy

$$a_{\rm peri} = a(1-e) = fR_{\rm Mars} \text{ , (S3)}$$

where f is smaller than 1. Thus, the debris ($e < 1$) re-impacts Mars during its single orbit. In contrast, if the debris particles have velocities larger than the escape velocity of Mars, they have hyperbolic orbits that escape Mars's gravity. Therefore, in our Monte Carlo simulations, we only considered a single orbit of the debris to assess the likelihood of the ejecta being transferred to a Martian moon, Phobos or Deimos.

It is also worth mentioning that for an oblique crater-forming impact the debris with small angle of velocity vector from the surface of the impacted planet has higher velocity than the

debris with an angle more perpendicular to the surface due to small energy damping during the collision with the surface. Thus, by the nature of a closed trajectory of a Keplerian orbit of debris, higher-velocity ejecta has a pericenter distance close to the radius of the impacted planet (f becomes closer to 1) and vice-versa.

S1.2. Minimum and maximum impact velocity of ejecta to the moons

Assuming the Martian moon has a circular orbit with its semi-major axis a_{moon}, the minimum relative velocity between the impact debris and the moon during their orbital crossing is established when the orbital plane of the impact debris is the same as that of the moon and the orbit of the debris is aligned to that of the moon at their apocenter distance ($e < 1$) in the prograde direction as

$$a(1+e) = a_{\mathrm{moon}}. \quad \text{(S4)}$$

Solving Eqs. (S3) and (S4) yields the unique set of orbital elements of the impact debris whose relative velocity to the moon is the minimum:

$$e = \frac{1 - \frac{f R_{\mathrm{Mars}}}{a_{\mathrm{moon}}}}{1 + \frac{f R_{\mathrm{Mars}}}{a_{\mathrm{moon}}}} \quad \text{(S5)}$$

$$a = \frac{1}{2}(a_{\mathrm{moon}} + f\, R_{\mathrm{Mars}}). \quad \text{(S6)}$$

The velocity of the impact debris at the apocenter is given by

$$v_{\mathrm{apo}} = v_K \sqrt{\left(\frac{1-e}{1+e}\right)} = \sqrt{\frac{GM}{a(1+e)}}\sqrt{1-e} = v_{\mathrm{moon}}\sqrt{1-e} \sim \begin{cases} 2\ [\mathrm{km/s}] \times \sqrt{1-e} & \text{for Phobos} \\ 1.35\ [\mathrm{km/s}] \times \sqrt{1-e} & \text{for Deimos} \end{cases}, \quad \text{(S7)}$$

where $v_{\mathrm{K}} = \sqrt{\frac{GM}{a}}$ is the Kepler velocity (G is the gravitational constant). The left panel of Figure S7 shows the minimum impact velocity, $v_{\mathrm{imp,min}} = v_{\mathrm{moon}} - v_{\mathrm{p,apo}}$, as a function of pericenter distance of the debris. For a pericenter distance of ~R_{Mars}, the impact velocity becomes ~550 m/s and it increases to ~950 m/s as the pericenter distance decreases to ~0.5R_{Mars}.

The maximum impact velocity would occur when the orbit of the debris is a hyperbolic orbit ($e > 1$). The velocity of a hyperbolic orbit at the moon's orbital distance to Mars can be written as

$$v_{\mathrm{p,hyper}} = \sqrt{GM_{\mathrm{Mars}}}\sqrt{\frac{2}{a_{\mathrm{moon}}} - \frac{1}{a_{\mathrm{p,hyper}}}}, \quad \text{(S8)}$$

where $a_{\mathrm{p,hyper}}$ is the semi-major axis of the debris given by

$$a_{\mathrm{p,hyper}} = \left(\frac{2}{R_{\mathrm{Mars}}} - \frac{v_{\mathrm{D,sur}}}{GM}\right)^{-1} \quad , \qquad \text{(S9)}$$

where $v_{\mathrm{D,sur}}$ is the velocity of the debris at the surface of Mars (just after the impact), which can be larger than the impact velocity. The right panel of Figure S7 shows the expected maximum impact velocity of hyperbolic debris with Martian moons, $v_{\mathrm{imp,moon}} = \sqrt{v_{\mathrm{p,hyper}}^2 + v_{\mathrm{moon}}^2}$, which can be larger than ~10 km/s.

S1.3. Favorable geometrical condition for impact ejecta to strike Phobos

In this subsection, we discuss the favorable geometrical condition of impact at the surface of Mars relative to the orbit of the Martian moons for the impact ejecta to reach the moon Phobos. The moon orbits almost on the equatorial plane of Mars. However, asteroidal impacts on Mars can occur anywhere at the surface of Mars. It is expected that collision between impact ejecta and Phobos would preferentially occur when Phobos is nearly above the impact point at the epoch of impact, because the orbital height of Phobos is only about ~1.6 times the Martian radius from the surface of Mars. The minimum time duration for the ejecta to reach Phobos is estimated as a function of ejection velocity by considering the vertical throw up motion as

$$\Delta t = \frac{v_{\mathrm{eje}}}{g_{\mathrm{M}}} = 1104\ [\mathrm{s}] \times \left(\frac{v_{\mathrm{eje}}}{4.1\ [\mathrm{km\ s^{-1}}]}\right)\left(\frac{g_{\mathrm{M}}}{3.711\ [\mathrm{m\ s^{-2}}]}\right), \qquad \text{(S10)}$$

where $g_M = 3.711$ [m/s^2] is the surface gravity of Mars and v_{eje} is the ejection velocity of the impact ejecta at the surface of Mars. The most probable ejection velocity for a strike to Mars is v_{eje} ~4.1 [km/s] (see Section S3). Using these values, we obtain ~1100 [s] to reach Phobos. During this time duration, the orbital phase of Phobos changes by only ~15 degrees. However, in reality, because the surface gravity decreases as the ejecta travels to a higher orbit and because the real orbit is likely a parabolic orbit, it takes more time to reach Phobos. The simulations show that a large fraction of the ejecta takes about ~2000–5000 [s] to reach Phobos (the phase of Phobos can change by ~26–66 degrees during this time period).

Here, to see clearly the effect of the relative equatorial location between the impact point at the surface of Mars and the orbital phase of Phobos on the transferred ejecta mass to Phobos, we use the data obtained from the impact forming the Zunil Crater (Zunil is located at the latitude of 7.7° N). Figure S8 shows the transferred ejecta mass against the relative equatorial location between the Phobos phase and Zunil Crater at the time of impact (Phobos phase − longitude of Zunil).

As we can see in Figure S8, moderate grazing impacts (impact angles of ~30–45 degrees) are the most efficient impacts to deliver impact ejecta to Phobos (Figure S8 also reveals the efficient mass delivery at these grazing impacts). The negative values in relative equatorial location indicate that Phobos is not above the impact point at the time of impact on the Martian surface, that is, Phobos is approaching a point above the impact point. As we discussed above, the phase

of Phobos can shift ~30 degrees between the launching of the ejecta from the Martian surface and the arrival of the ejecta on Phobos, and this is seen as a discontinuous increase of the mass transferred at around −30 degrees in relative equatorial location in Figure S8.

S2. Mass transfer from Mars to Martian moon(s)

S2.1. Impact history of Mars

Throughout the history of solar system evolution, the planets have experienced continuous impact bombardment. Hartmann (2005)[18] updated the Martian crater isochron. In this work, we used this new Martian crater isochron of the past 1 Gyr to derive that of the past 500 Myr (their Table 2). We can assume that the asteroidal flux has been almost constant since 3 Ga (their Table 2); thus, we simply divided the value of the past 1 Gyr of their crater isochron by a factor of 2 to derive that of the past 500 Myr (Figure S9). In order for the impact ejecta to penetrate the Martian atmosphere, the size of the crater needs to be larger than ~2 km in diameter[44]. The cumulative number of craters within the past 500 Myr is well fitted by the following exponential relationship:

$$N(D > 2\ \mathrm{km}) = \alpha D^{-\beta}\ , \quad \text{(S11)}$$

where N (D > 2 km) is the cumulative number of craters on the Martian surface with diameter D larger than 2 km (solid line in Figure S9). By fitting the date obtained from Hartman's isochron (Figure S9), we derived α = 41325 and β = 1.9096.

Using the Bootstrap method[53], our 30,000 Monte Carlo runs can provide an averaged crater size to delivered mass relationship (Figure 1 in the main text) as

$$M_{\mathrm{transport}}(D) = \gamma D^{\varepsilon}\ , \quad \text{(S12)}$$

where $M_{\mathrm{transport}}$ is the mass of Martian impact debris that is delivered to Phobos by a crater-forming event whose crater diameter is D. We found that $\gamma = 10^{2.34 \pm 0.62}$ and $\varepsilon = 3.19$.

To calculate the total transferred mass of impact debris to Martian moon(s) during the past 500 Myr, we simply needed to multiply and integrate the above two fitting equations. To obtain the "statistical" value where the number of impacts in the Martian isochron is expected to be larger than 10 (D < 100 km; see Figure S9), we integrated as follows:

$$\begin{aligned} M_{\mathrm{transport,total}} &= \int_{D_{\min}}^{D_{\max}} M_{\mathrm{transport}}(D) \left(\frac{dN(D)}{dD}\right) dD \\ &= \frac{\alpha\beta\gamma}{\epsilon-\beta}\left[D_{\max}^{\epsilon-\beta} - D_{\min}^{\epsilon-\beta}\right] \sim \frac{\alpha\beta\gamma}{\epsilon-\beta} D_{\max}^{\epsilon-\beta}\ , \quad \text{(S13)} \end{aligned}$$

where we used $D_{\max}$ = 100 km and $D_{\min}$ = 2 km. We obtain $M_{\mathrm{transport,total}} = 4.9 \times 10^9$ kg for the statistical value of the past 500 Myr. To find the "stochastic" value where the number of impacts is expected to be less than 10 (D > 100 km), we used $D_{\max}$ = 300 km and $D_{\min}$ = 100 km.

S2.2. Comparison to previous works

Figure S10 shows the averaged total transported rock from the five recent craters on Mars, and the sum of the average total mass was found to be ~4.7×10^8 kg for Phobos and ~9.1×10^6 kg for Deimos. If uniform mixing of the Martian rocks with the regolith on Phobos is assumed to be one-meter-depth[20,21], the mixing ratio of the Martian materials to Phobos regolith is estimated to be ~150 ppm. By following the same procedure, we estimate that the mixing ratio of the Martian materials to Deimos regolith is ~9 ppm. This mixing ratio of Phobos is ~2 orders of magnitude larger than the previous estimation of ~2 ppm obtained by Chappaz et al. (2013)[20].

This significant upward revision is mainly due to the consideration of the Mojave-forming event. Previous studies neglected this event and considered only three smaller large craters (Zunil, McMurdo, and Tooting). This was done because the formation age of Mojave had not been constrained at the time when the previous papers were researched. The age of Mojave was recently determined as 3–5 Ma based on a crater chronology model and the recently obtained high-resolution images around Mojave Crater[17].

In addition, because we used realistic impact conditions of the grazing impacts and used 3D direct SPH simulations to obtain the initial position/velocity distributions of the impact ejecta, the total ejection mass was increased by a factor of ~4.5 at an impact angle of 45 degrees compared to an impact angle of 90 degrees (Figure S11). Previous works[20,21] used the point-source approximation, which cannot be applied to the launching of high-speed ejecta[34,44,46], and the ejecta mass distribution as a function of the ejection velocity was obtained from an analytical estimation (cone-shape distribution model). Additionally, in this study, the mass distribution of the ejecta as a function of the ejection velocity was increased by several factors at the high impact probability regime around $v_{\rm eje} \sim 4.2$ km/s (Figure S11). Due to these two effects, the net mass transferred to Phobos was increased by 1 order for a single crater-forming event compared to the results of the previous works[20,21].

S3. Impact probability of the ejecta with Martian moons

Numerous impact ejecta launched from the surface of Mars were assumed to be randomly distributed into the space and to cross the sphere whose radius is the semi-major axis of the Martian moons. Thus, the average impact probability with Phobos and Deimos can be estimated by considering the relative area of cross sections of Martian moons to surface area of the sphere as

$$P \sim \frac{1\ (\text{for e} > 1)\ \text{or}\ 2\ (\text{for e} < 1) \times \pi R_{\text{moon}}^2}{4\pi a_{\text{moon}}^2}$$
$$= \begin{cases} 3.44 \times 10^{-7} (\text{for e} > 1)\ \text{or}\ 6.88 \times 10^{-7}\ \ (\text{for e} < 1)\ \ \text{for Phobos} \\ 1.63 \times 10^{-8}\ (\text{for e} > 1)\ \text{or}\ 3.27 \times 10^{-8}\ \ (\text{for e} < 1)\ \ \text{for Deimos} \end{cases}, \quad \text{(S14)}$$

where $R_{\rm moon}$ is the radius of Martian moon. The factors of 1 or 2 are due to the nature of the closed (e < 1) or open (e >1) Keplerian orbits. When e < 1, orbital crossing between the ejecta and the moon can occur at two epochs during a single orbit of the ejecta, when the ejecta travels

from its pericenter to apocenter and when it travels from its apocenter to pericenter. In contrast, when $e > 1$, impact occurs only at a single time when the ejecta travels from the surface of Mars to infinity (outside the gravity field of Mars).

S3.1. Impact probability for Phobos

Figure S12 shows the impact probabilities for Phobos obtained in our results from the two-body orbital calculation for a variety of crater-forming events. The cumulative impact probability as a function of the ejection velocity is shown by the dashed lines in Figure S12. The impact probability at a specific ejection velocity is shown by the solid lines in Figure S12. The sudden jump of probability at ~5 km/s (the difference is by a factor of ~2) is due to the nature of the Keplerian orbits. As explained above, if $v_{eje} > 5$ km/s, the orbit of the ejecta is a hyperbolic orbit and impact with the moon occurs only when the ejecta is traveling from the surface to infinity; however, when $v_{eje} < 5$ km/s, the orbit is an eccentric orbit around Mars and the ejecta can collide with the moon at "two epochs" in a single orbit, when traveling from the launching point to the apocenter and when traveling from the apocenter back to the pericenter.

In the fully randomized case, the total impact probability is $\sim 6 \times 10^{-7}$ (dashed line in Figure S12), which is consistent with the analytical argument (Eq. (S14)). Note that the overall magnitude and behavior of the impact probability (especially for $v_{eje} > 5$ km/s) are consistent with those of the previous work[20]; however, the previous work considered only the specific cases of cone-shape ejection with limited ejection angles and velocities. Differently, our results include all possible types of ejection with a variety of ejection angles and ejection velocities resulting from various crater-forming events by using direct numerical calculations (SPH impact simulations and analytical orbital integration).

The impact probability depends on the latitude of the crater-forming event. For example, the probability is similar at Mojave, Corinto, and Zunil because their locations are near the Martian equator. Also, for the near-equator crater-forming events, the impact results in high ejection velocity and the ejecta has a hyperbolic orbit ($v_{eje} > 5$ km/s). In contrast, the impact probability for McMurdo with a latitude close to the south pole of Mars is limited within a very narrow window of ejection velocity ($v_{eje} \sim 4$–5 km/s). If the ejecta velocity is higher than ~5 km/s, the ejecta orbits are hyperbolic (ballistic). From a high latitude such as that of McMurdo, this high-speed ejecta can reach the orbital plane of Martian moons (equatorial plane of Mars) at only much farther distance from Mars, i.e., if you draw a straight line parallel to the local Mars surface at sufficiently high latitude, the line only crosses the orbital plane of Martian moon far beyond the orbit of Phobos.

S3.2. Impact probability for Deimos

Figure S13 shows the impact probabilities of the ejecta from the different craters for Deimos. Compared to the case of Phobos (Figure S12), the minimum velocity required to reach Deimos is shifted to the higher ejection velocity of ~4.5 km/s and the peaks of the probability are also shifted to a slightly higher value of ~4.6 km/s. The average impact probability is $\sim 1.5 \times 10^{-8}$, which is consistent with the analytical estimation (Eq. (S14)).

S4. Impact velocity to Martian moons

The distribution of impact velocity of the ejecta at the time of collision with Martian moons is important not only for understanding the surface erosion processes of the moons but also for understanding the degree of impact sterilization during the delivery of Martian materials to the moons[54,55].

S4.1. Distribution of impact velocity on Phobos

Figure S14 shows the cumulative fraction of the impact velocity of the ejecta at the surface of Phobos. Most of the ejecta has impact velocities of ~1–20 km/s, and only a small fraction of the ejecta has impact velocities smaller than ~2 km/s. The outcome depends strongly on the latitude of the crater-forming event. For example, Mojave and Tooting show a similar distribution, but McMurdo shows a distribution largely different from the others. As McMurdo is located close to the south pole of Mars, the orbits of the ejecta launched from McMurdo and reaching Phobos always cross the orbit of Phobos. Thus, the impact velocity of such ejecta is larger than the orbital velocity of Phobos (~2 km/s). Also, as discussed in Section 1, when the ejecta has a hyperbolic orbit from McMurdo ($v_{\mathrm{eje}} > \sim 5$ km/s and a corresponding orbital velocity at the location of Phobos (impact velocity) of $v_{\mathrm{imp}} > \sim 3$ km/s), the ejecta cannot reach Phobos; thus, the impact velocity is limited to ~2–3 km/s for the McMurdo-forming event. In the fully randomized case, the impact velocity to Phobos is ~1–20 km/s, as seen in the other cases. The sudden change of the slope of the distribution at around $v_{\mathrm{imp}} \sim 3$ km/s is due to the change of orbit from an eccentric orbit ($v_{\mathrm{eje}} < 5$ km/s) to a hyperbolic orbit ($v_{\mathrm{eje}} > 5$ km/s).

S4.2. Distribution of impact velocity on Deimos

Figure S15 shows the distribution of the impact velocity of the ejecta to Deimos. The overall trends are similar to those in the case of Phobos (Figure S14), although the minimum impact velocity is shifted to lower values. This shift occurs because the orbital distance of Deimos from Mars is larger than that of Phobos. Thus, the relative velocity becomes smaller because the orbital velocity of the moon becomes smaller as the distance from Mars becomes larger. The change of the slope of the distribution at around ~1.8 km/s clearly seen for the cases of Mojave, Tooting, and Corinto is due to the change of orbit of ejecta from an eccentric orbit to a hyperbolic orbit.

S5. Localization of impact on Martian moons

When the debris has $e < 1$ and $i \sim 0$, where i is the inclination with respect to the Martian equator, as $v_{\mathrm{Phobos}} > v_{\mathrm{apo}}$ (Eq. (S7)), an impact with smallest impact velocity occurs on the leading side of the moon in the way that the moon catches up to the orbit of the ejecta particle. In contrast, on the trailing side (and especially the far side with respect to Mars), it is very difficult for ejecta to collide with the moon with an impact velocity smaller than the orbital velocity of the moon (~2 km/s for Phobos and ~1.35 km/s for Deimos) because the velocity of the ejecta at the apocenter is smaller than the moon's orbital velocity (Eq. (S7)); thus, the ejecta cannot catch up from the trailing side of the moon.

When the debris has $e > 1$ and $i \sim 0$, the ejecta has a hyperbolic orbit (ballistic orbit). A hyperbolic orbit is not closed and the ejecta does not return to Mars. Hence, impact with hyperbolic ejecta occurs only on the near side of Phobos and Deimos with respect to Mars.

S5.1. Local impact probability on Martian moons

In this subsection, we discuss the regional difference of the impact conditions of the ejecta on the surface of Martian moons. Both Phobos and Deimos are tidally locked, and the local impact condition may differ at the near/far sides or leading/trailing sides of the moons. We define the local impact probability per unit area as

$$P_{\mathrm{col,local}} = \frac{N_{\mathrm{coll}}}{\mathrm{N}_{\mathrm{coll,tot}} \times dS} \ , \qquad \text{(S15)}$$

where N_{coll} is the number of local collisions at longitude φ and latitude θ_{lat} of the moon, $N_{\mathrm{col,tot}}$ is the total number of collisions of the debris with the moon, and $dS = R_{\mathrm{moon}}^2 \, \Delta\varphi\Delta\theta_{\mathrm{lat}} \sin\theta_{\mathrm{lat}}$ is the local surface area of the moon, where R_{moon} is the average radius of the moon.

S5.2. Impact localization on Phobos

Figure S16 shows the local impact probability for different areas on the surface of Phobos. The probability depends strongly on the local area of the moons. The results also change according to the different crater-forming events on Mars. When a crater-forming event occurs in the northern hemisphere of Mars (e.g., McMurdo), impact between the debris and the moon rarely occurs near the polar region of the opposite hemisphere of the moon and vice-versa. When a crater-forming event occurs in the near-equatorial region of Mars (e.g., Mojave, Tooting, Corinto), a longitudinal dependence also appears because the ejecta that strikes the moon has a typical orbit near the orbital plane of the moon. In the case of Zunil, because the Zunil-forming impactor struck Mars from the east–northeast, most of the ejecta has a retrograde orbit with respect to Phobos and Deimos. Additionally, as the Zunil Crater is located slightly above the Martian equator (in the northern hemisphere of Mars), the ejecta is initially launched from above the Martian equatorial plane and travels to below the equatorial plane. Thus, only a small fraction of the ejecta strikes Phobos when travelling to its apocenter. In contrast, the ejecta crosses the equatorial plane when travelling back to Mars, and more ejecta collides with Phobos during this epoch. In the case of McMurdo, located near the south pole of Mars, the ejecta originated from McMurdo showers Phobos from the direction of the south pole of Phobos. A longitudinal dependence is not clearly seen, but a latitudinal dependence appears clearly. In the fully randomized case (Figure S16, right bottom), the longitudinal dependence becomes obscure compared to the case of the near-equatorial craters because the ejecta is launched from everywhere from the surface of Mars.

Figure S17 is similar to Figure S16, but it considers only ejecta with impact velocities smaller than 2 km/s. Compared to the all-ejecta case (Figure S16), the longitudinal dependences are much more clearly seen in Figure S17. As discussed in Section 2.3, low-velocity collisions (v_{imp} < 2 km/s) rarely occur at the far side and the trailing side of Phobos due to the orbital properties of the ejecta launched from near the equator. In the case of McMurdo, an impact with impact velocity lower than 2 km/s never occurs due to the orbital crossing of the ejecta and Phobos (the orbital velocity of Phobos is ~2 km/s).

S5.3. Impact localization on Deimos

Figures S18 and S19 show the local impact probability for the case of Deimos. Figure S18 considers all the ejecta, and the results are similar to the cases of Phobos (Figure S16). When we consider only the ejecta with impact velocities smaller than 2 km/s (Figure S19), the locations of impact change compared to the cases of Phobos (Figure S17) because the orbital velocity of Deimos is different from that of Phobos. Note that the resolutions of Figures S18 and S19 are not as good as those of Figures S16 and S17 because the number of ejecta colliding with Deimos is much smaller than that colliding with Phobos.

S6. Impact flux of natural asteroids

Natural background asteroid impacts take place both on Mars and on its moons. Here we calculate the impact flux of natural asteroids on Mars, Phobos and Deimos for the past 500 Myr. For this, we calculate the impactor size-frequency distributions (SFD) to Mars from craters on Mars using Hartmann isochrons[18]. The conversion from the crater size to impactor size is described in Kurosawa et al. (2019)[54]. For the calculation, we assume that impacts occur at a velocity of 14 km/s and an angle of 45 degrees. The densities of the impactor and the surface materials of Mars are both assumed to be 2.7×10^3 kg/m^3. Figure S20 shows the impactor SFDs for Mars, Phobos, and Deimos (dots). We fit the SFD of impactor to Mars using two power-law functions (one for $D_\mathrm{p} < 0.1$ km and the other for $D_\mathrm{p} > 0.1$ km, both shown by dashed lines in Figure S20). The impactor SFDs for Phobos and Deimos are approximated by the ratios of the cross sections of the moons to Mars. To calculate the cumulative total masses of natural asteroidal impacts to Mars, Phobos and Deimos ($M_\mathrm{natural,imp,total}$), we integrate the following equation for Mars, Phobos and Deimos:

$$M_\mathrm{natural,imp,tot} = \int_{D_\mathrm{p,min}}^{D_\mathrm{p,max}} m_\mathrm{imp}(D_\mathrm{p}) \frac{dN}{dD_\mathrm{p}} dD_\mathrm{p} \,,$$

where N is the fitted impactor SFDs for Mars, Phobos, or Deimos; dN is the increment of the number of bodies in the interval $(D_\mathrm{p}, D_\mathrm{p} + dD_\mathrm{p})$; and $m_\mathrm{imp}(D_p)$ is the impactor mass whose diameter is D_p. $D_\mathrm{p,min} = 10^{-4}$ km is the minimum size of the impactor considered here. $D_\mathrm{p,max}$ is the diameter whose SFDs equal 1 for Mars, Phobos, or Deimos. Thick lines in Figure S20 show the cumulative mass of natural impacts for Mars, Phobos, and Deimos. For Phobos, a total of more than ~10^9 kg of impacts occurred over the past 500 Myr. This mass is larger than that delivered from Mars, as impact ejecta, by a single event; the mass of Martian ejecta from the largest Mojave-forming event is ~10^8 kg. More detailed and complex study of the impact processes and their subsequent dynamics on the moons are necessary to make more precise estimates of mass accreted by them; this is beyond the scope of this paper. Importantly, our main results—those regarding Martian ejecta embedded in the regolith of Martian moons—are not affected by the number of naturally occurring impacts on the Martian moons.

References (in the Supplementary Materials**):**

32. Lucy, L.B. A numerical approach to the testing of the fission hypothesis. *Astron. J.* **82**, 1013-1024 (1977).
33. Monaghan, J.J. Smoothed particle hydrodynamics. *Annu. Rev. Astron. Astrophys.* **30**, 543-574 (1992).
34. Kurosawa, K., Okamoto, T. and Genda, H. Hydrocode modeling of the spallation process during hypervelocity impacts: Implications for the ejection of Martian meteorites. *Icarus* **301**, 219-234 (2018).
35. Tillotson, J.H. Metallic Equations of State for Hypervelocity Impact. *General Atomic Report* GA-3216 (1962).
36. Allen, R.T. Equations of state of rocks and minerals. General Dynamics, General Atomic Division (1967).
37. Johnson, B.C. & Melosh, H.J. Formation of melt droplets, melt fragments, and accretionary impact lapilli during a hypervelocity impact. *Icarus* **228**, 347-363 (2013).
38. Parkos, D., Pikus, A., Alexeenko, A. & Melosh, H.J. HCN production via impact ejecta reentry during the late heavy bombardment. *J. Geophys. Res.* **123**, 892-909 (2018).
39. Ito, T. & Malhotra, R. Dynamical transport of asteroid fragments from the n6 resonance. *Advances in Space Research* **38**, 817-825 (2006).
40. Shoemaker, E.M. Interpretation of lunar craters. In Physics and Astronomy of the Moon, edited by Z. Kopal, pp. 283–359, Academic, San Diego, Calif (1962).
41. Schmidt, R.M. & Housen, K.R. Some recent advances in the scaling of impact and explosion cratering. *International Journal of Impact Engineering* **5**, 543-560 (1987).
42. McKinnon, W.B., Chapman, C.R. & Housen, K.R. Cratering of the Uranian satellites. In Uranus, edited by Bergstralh, J.T., Miner, E.D., and Matthews, M.S., University of Arizona Press, Tucson, 629-692 (1991).
43. Pike, R.J. Geomorphology of impact craters on Mercury. In: Mercury, pp. 165–273. University of Arizona Press, Tucson (1988).
44. Artemieva, N. & Ivanov, B. Launch of meteorites in oblique impacts. *Icarus* **171**, 84–101 (2004).
45. Maxwell, D.E. Simple Z model of cratering, ejection, and the overturned flap. In Impact and Explosion Cratering, edited by D.J. Roddy, R.O. Pepin, and R.B. Merrill, pp. 1003–1008, Pergamon Press, New York (1977).
46. Melosh, H.J. Impact Cratering: A Geologic Process. Oxford University Press, New York (1989).
47. Tornabene, L.L. *et al*. Identification of large (2–10 km) rayed craters on Mars in THEMIS thermal infrared images: Implications for possible Martian meteorite source regions. Journal of Geophysical Research 111, E10006 (2006).
48. McEwen, A.S. *et al*. The rayed crater Zunil and interpretations of small impact craters on Mars. *Icarus* **176**, 351-381 (2005).
49. Preblich, B.S., McEwen, A.S. & Studer, D.M. Mapping rays and secondary craters from the Martian crater Zunil. *J. Geophys. Res*. **112**, E05006 (2007).
50. Murray, C.D. & Dermott, S.F. Solar System Dynamics, Cambridge University Press, Cambridge, UK (1999).
51. Hyodo, R., Ohtsuki, K. & Takeda, T. Formation of multiple-satellite systems from low-mass circumplanetary particle disks. *Astrophys. J.* **799**, 40 (2015).

52. Hyodo R. & Charnoz S. Dynamical evolution of the debris disk after a satellite catastrophic disruption around Saturn. *Astron. J.* **154**, 34 (2017).
53. Efron, B. & Tibshirani, R.J. An Introduction to the Bootstrap. CRC Press, Boca Raton, FL 1(1993).
54. Kurosawa K. *et al.* Assessment of the probability of microbial contamination for sample return from Martian moons II: The fate of microbes on Martian moons. *Life Sciences in Space Research*; 10.1016/j.lssr.2019.07.006 (2019).
55. Fujita K. *et al.* Assessment of microbial contamination probability for sample return from Martian moons I: The departure of the microbes from Martian surface. *Life Sciences in Space Research*; 10.1016/j.lssr.2019.07.009 (2019).

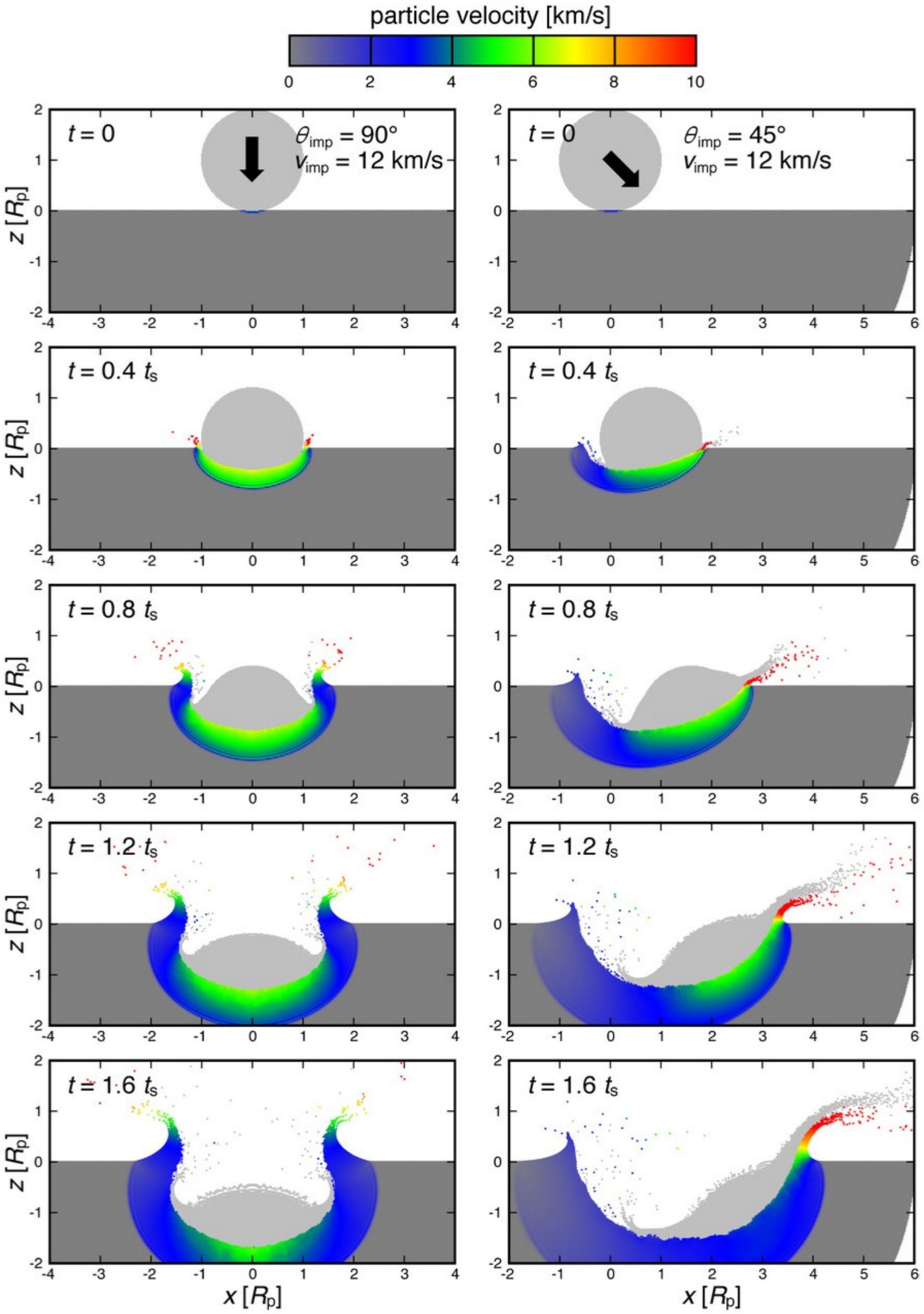

Figure S1. Snapshots of two example impacts with v_{imp} = 12 km/s for the case of a head-on impact (left panels) and a 45-degree impact (right panels). The color contours represent temporal particle velocity. All lengths are normalized by the projectile radius (R_{p}). The contact between the projectile and the target occurs at the origin. Only SPH particles in a thin cross section with y = −0.02 R_{p} to 0.02 R_{p} are plotted. Time (t) is normalized by the characteristic time for projectile penetration $t_{\mathrm{s}} = 2R_{\mathrm{p}}/v_{\mathrm{imp}}$. Projectile materials are shown in light gray.

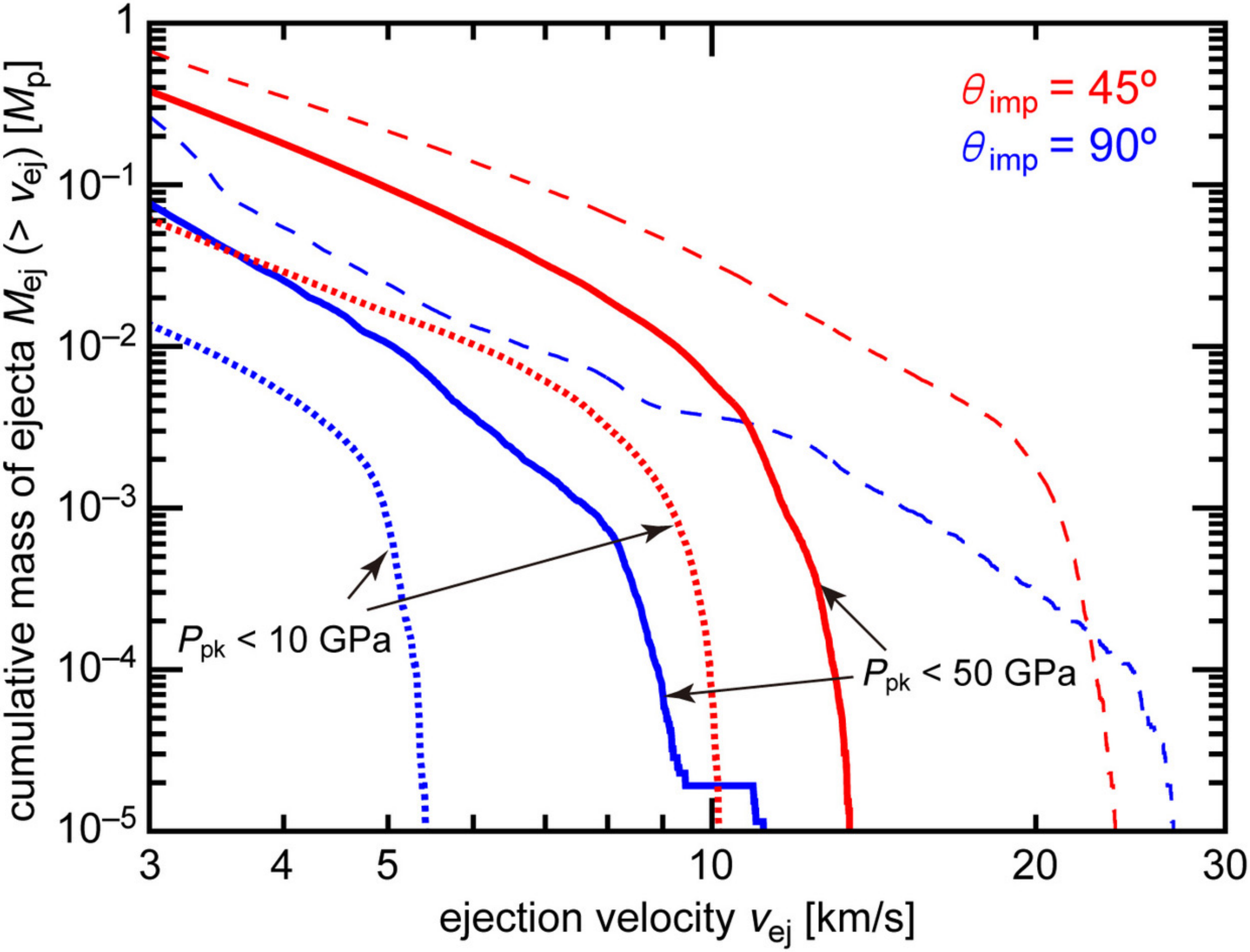

Figure S2. The cumulative mass of ejecta (M_{ej} (> v_{ej})) as a function of particle velocity (v_{ej}). Blue and red lines represent head-on impact and 45-degree impact with an impact velocity of 12 km/s, respectively. Projectile SPH particles are not included in this analysis. Thick solid lines and dotted lines represent the cumulative mass of SPH particles with peak pressures below 50 GPa and 10 GPa, respectively. Dashed lines represent the total cumulative mass.

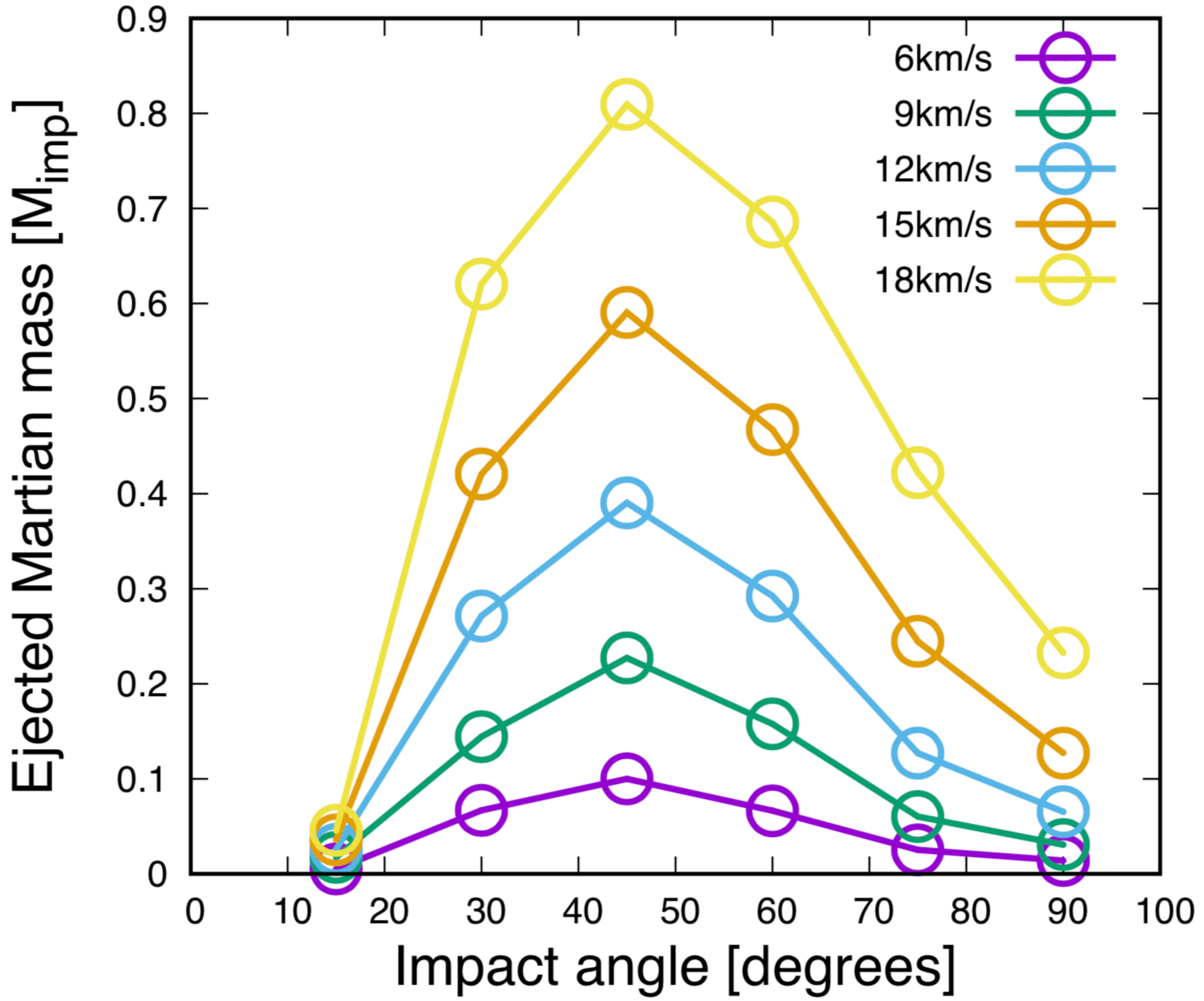

Figure S3. Ejected Martian mass whose ejection velocity is larger than 3.8 km/s normalized by the impactor mass for various impact angles and velocities.

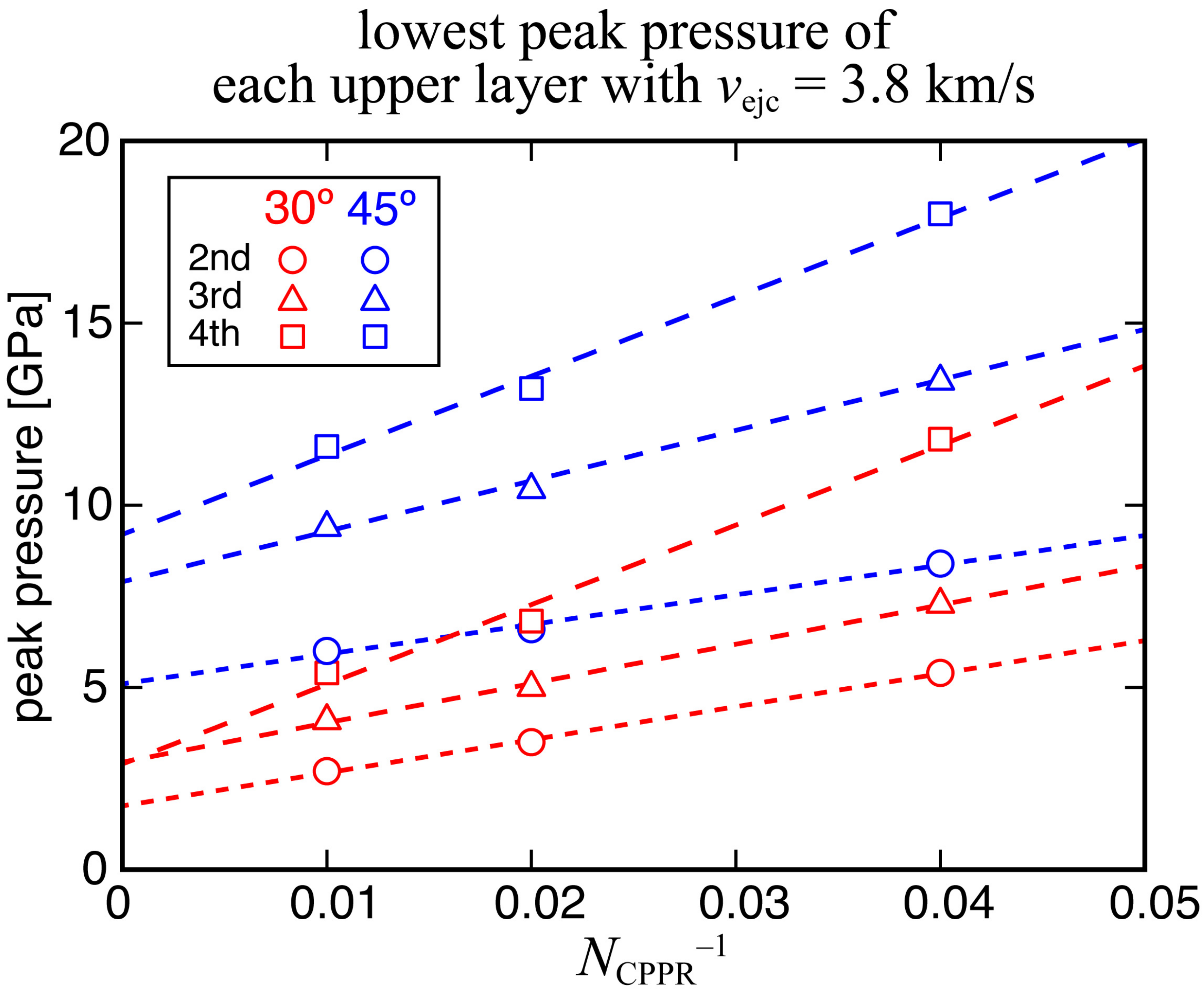

Figure S4. The lowest peak pressure of each upper layer from the surface of Mars whose ejection velocity is 3.8 km/s for the case of θ = 30 (red) and 45 degrees (blue). The points are obtained from our smooth-particle hydrodynamics (SPH) simulations whose resolutions are 25 particles per projectile radius (PPRP), 50 PPRP and 100 PPRP. The dashed lines are linear fittings to the SPH results by the least squares method. The intersection points between the straight lines and the left Y-axis are those at infinite spatial resolutions.

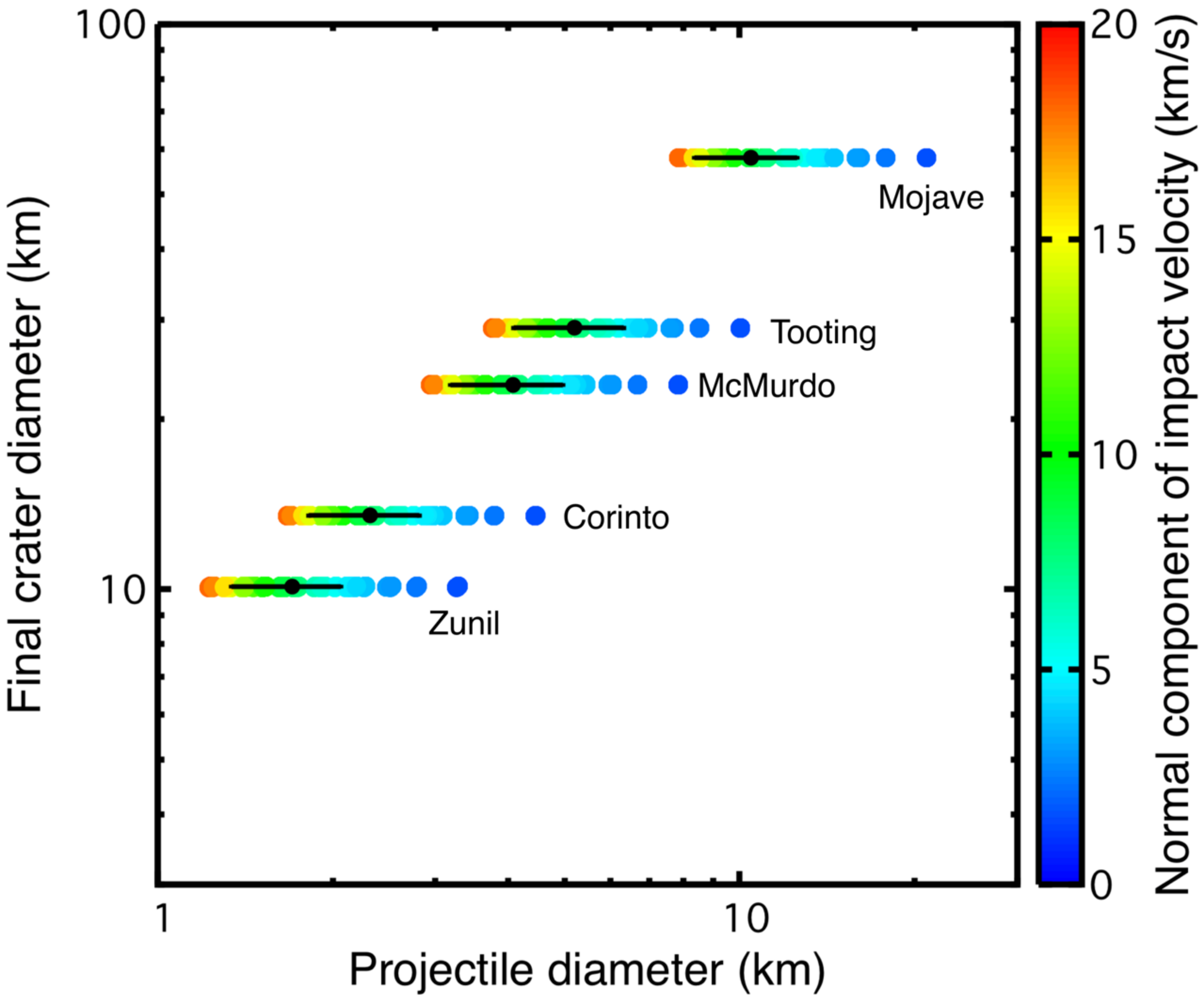

Figure S5. The calculated final crater diameter as a function of projectile diameter. The color scale shows the normal component of impact velocity. The name of each crater is shown in the figure. The averaged projectile diameters and their standard deviations are shown in the figure as the black filled circles with error bars.

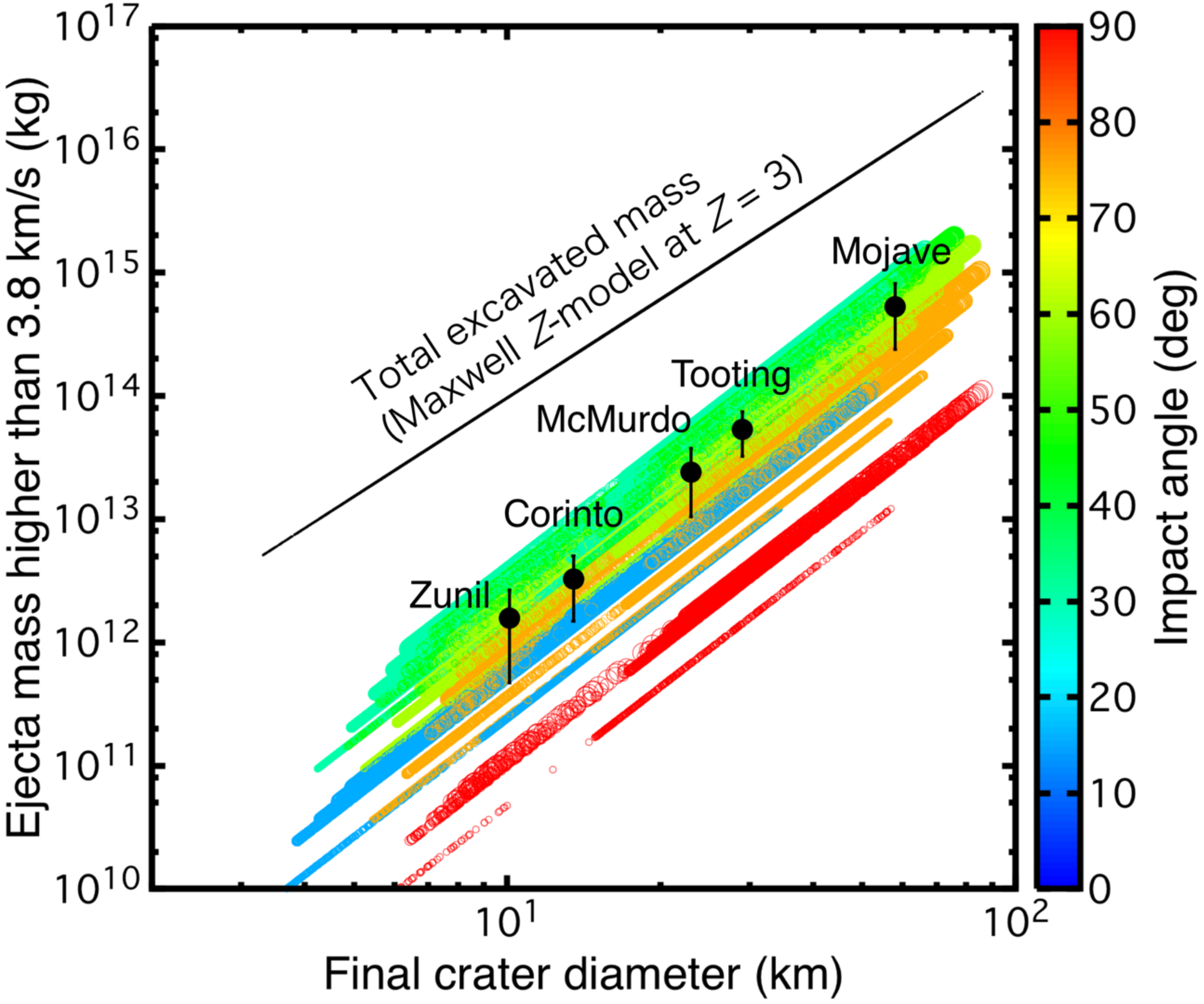

Figure S6. The masses of high-speed ejecta at velocities higher than 3.8 km/s as a function of the final crater diameter. The color scale and point size indicate the impact angle and velocity, respectively. The five craters are shown as black filled circles with error bars.

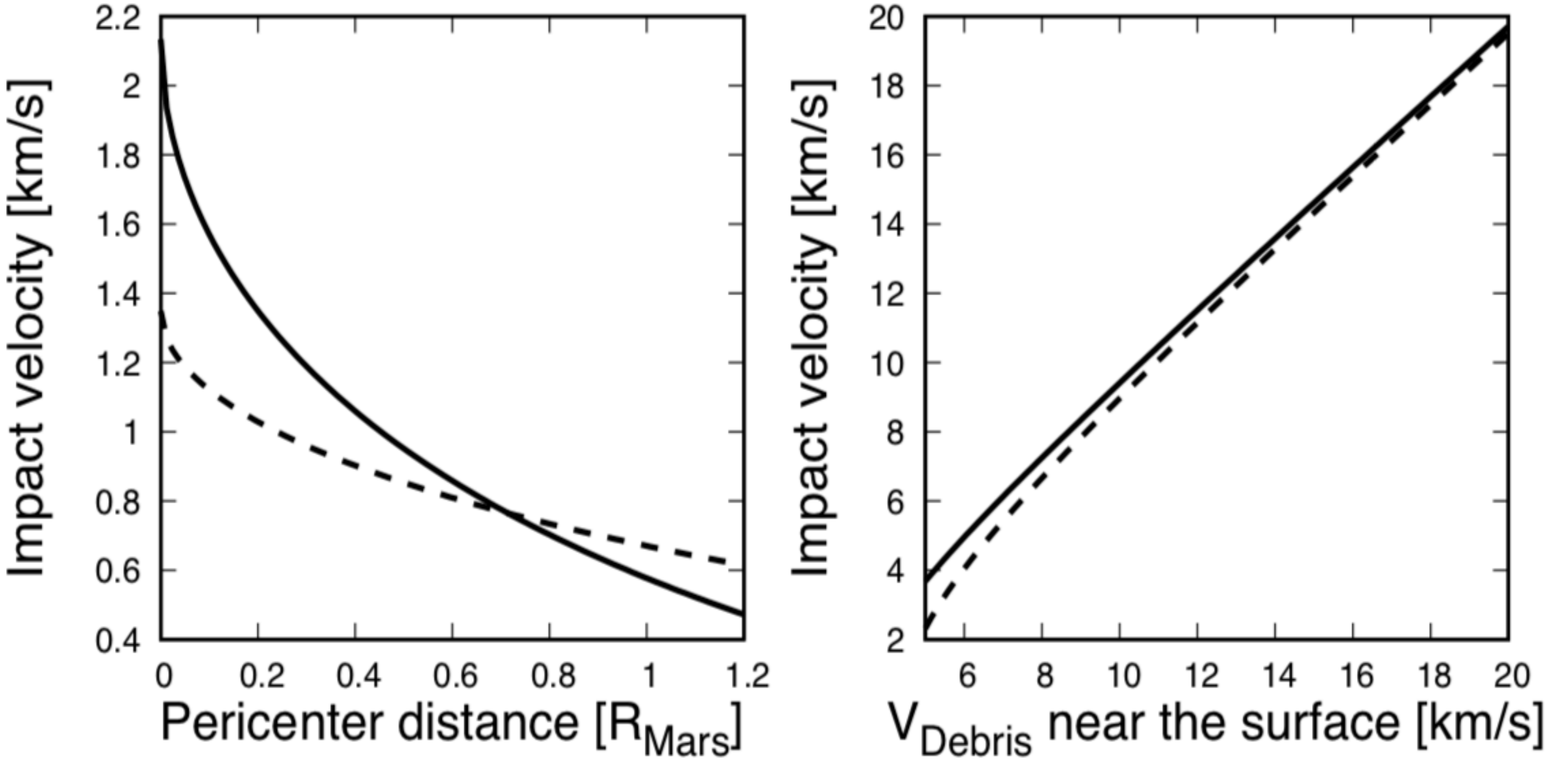

Figure S7. Minimum (left panel for eccentric orbits) and maximum (right panel for hyperbolic orbits) impact velocities of the debris on Phobos (solid line) and on Deimos (dashed line) as a function of their pericenter distance (left panel) and velocity of debris near the surface (right panel), respectively.

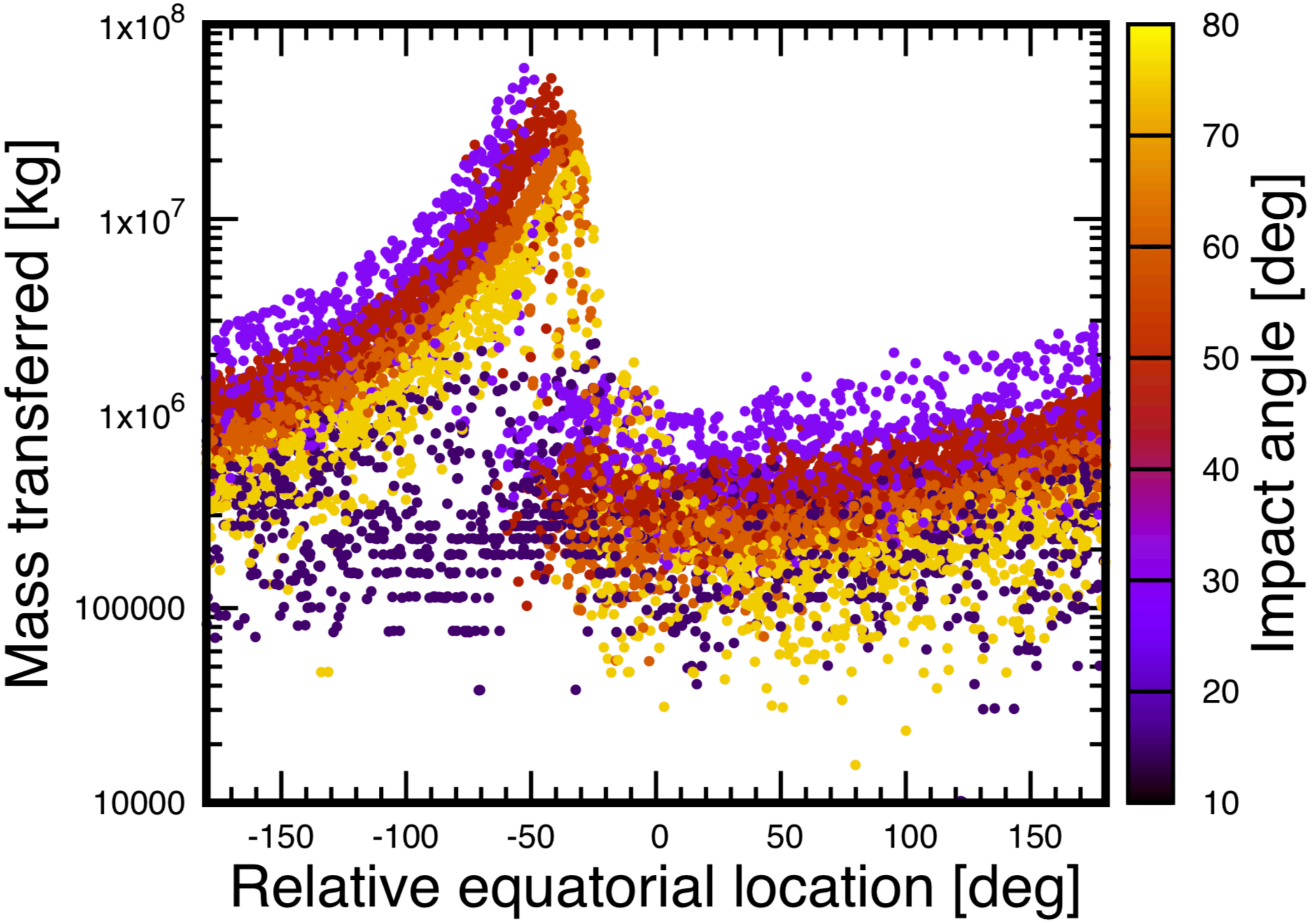

Figure S8. Transferred mass against relative equatorial location (Phobos phase – longitude of Zunil) in degrees. The color contour represents the impact angle to the surface of Mars.

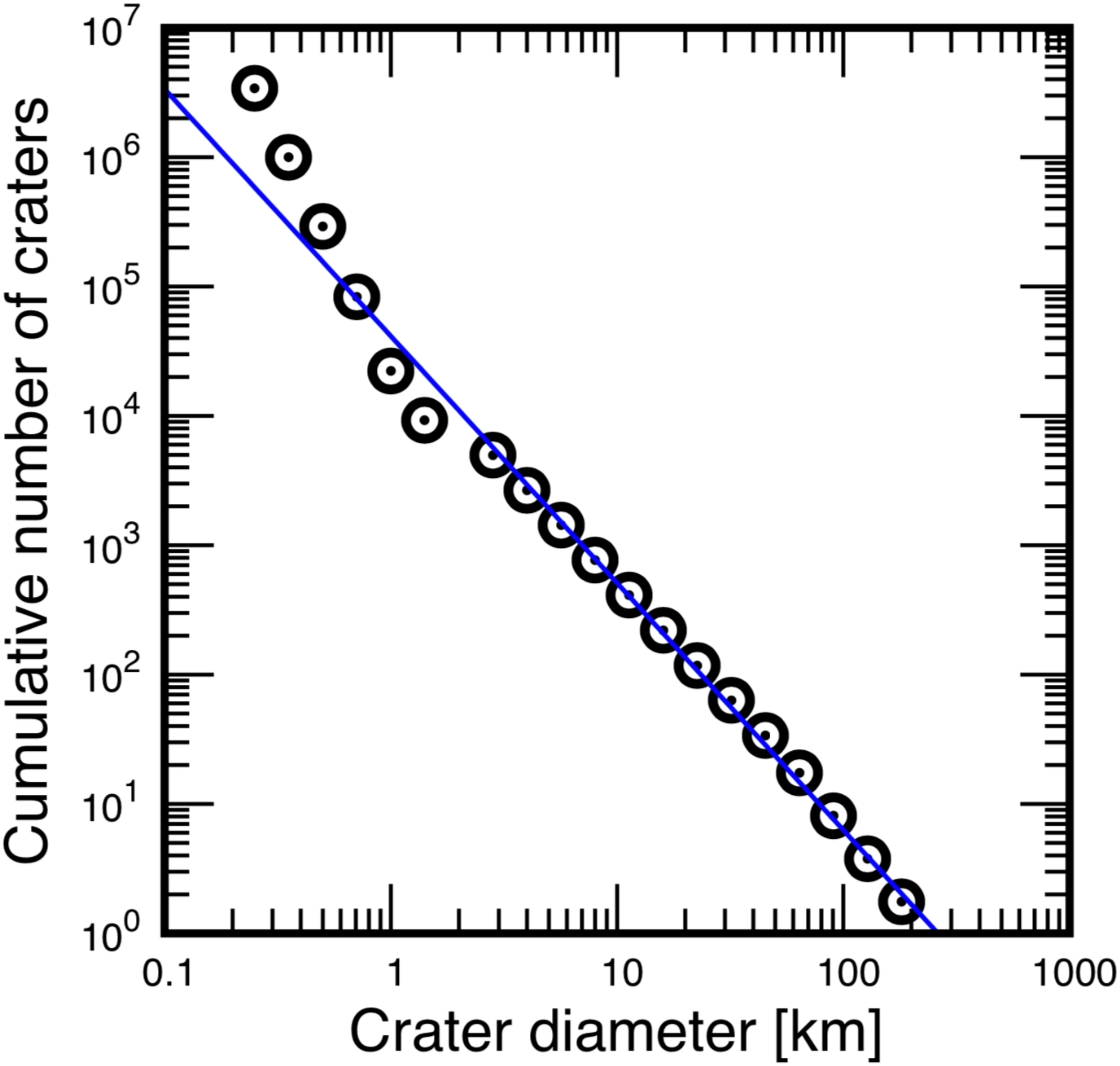

Figure S9. Cumulative number of craters on the Martian surface with age less than 500 Myr obtained from the isochron of Hartmann (2005). The blue line represents a fitted line for craters whose diameter is larger than 2 km.

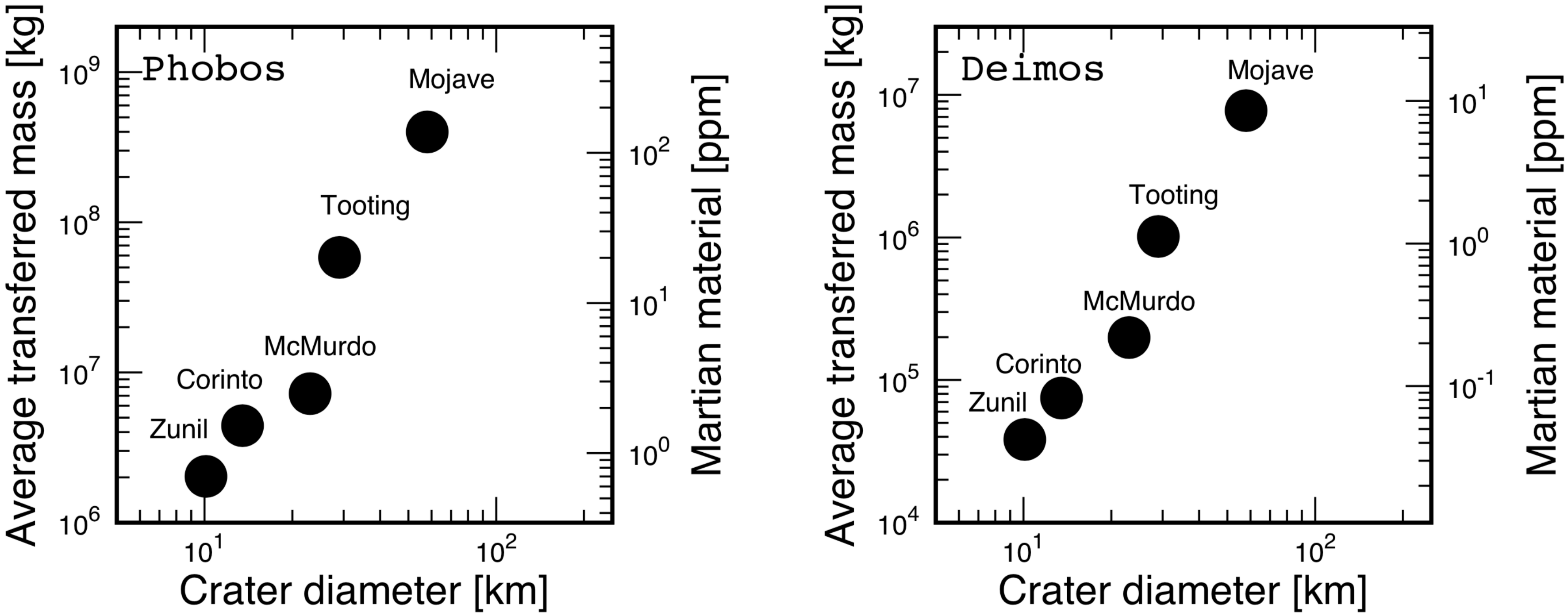

Figure S10. Average transferred mass from Mars to Phobos (left) and Deimos (right) as a function of crater diameter for the five largest recent craters. Right y-axis represents the mixing ratio of the transferred mass within 1-m surface regolith of Phobos and Deimos.

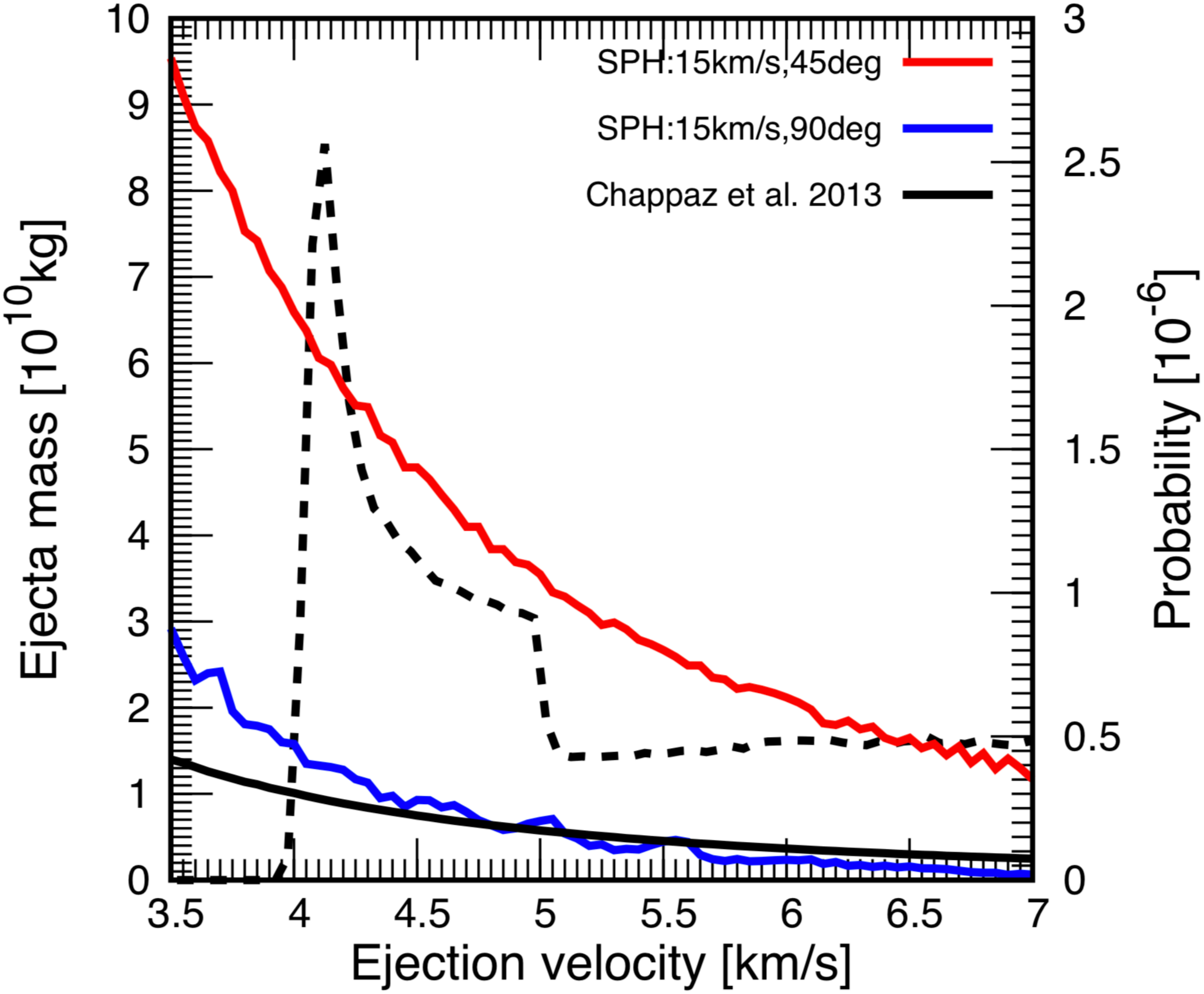

Figure S11. Ejecta mass against ejection velocity. Red and blue solid lines represent results obtained from SPH simulations for 45- and 90-degree impacts, respectively. Black solid line shows the result obtained from the analytical model (cone-shaped model) in Chappaz e al. (2013). Black dashed line is the impact probability against ejection velocity.

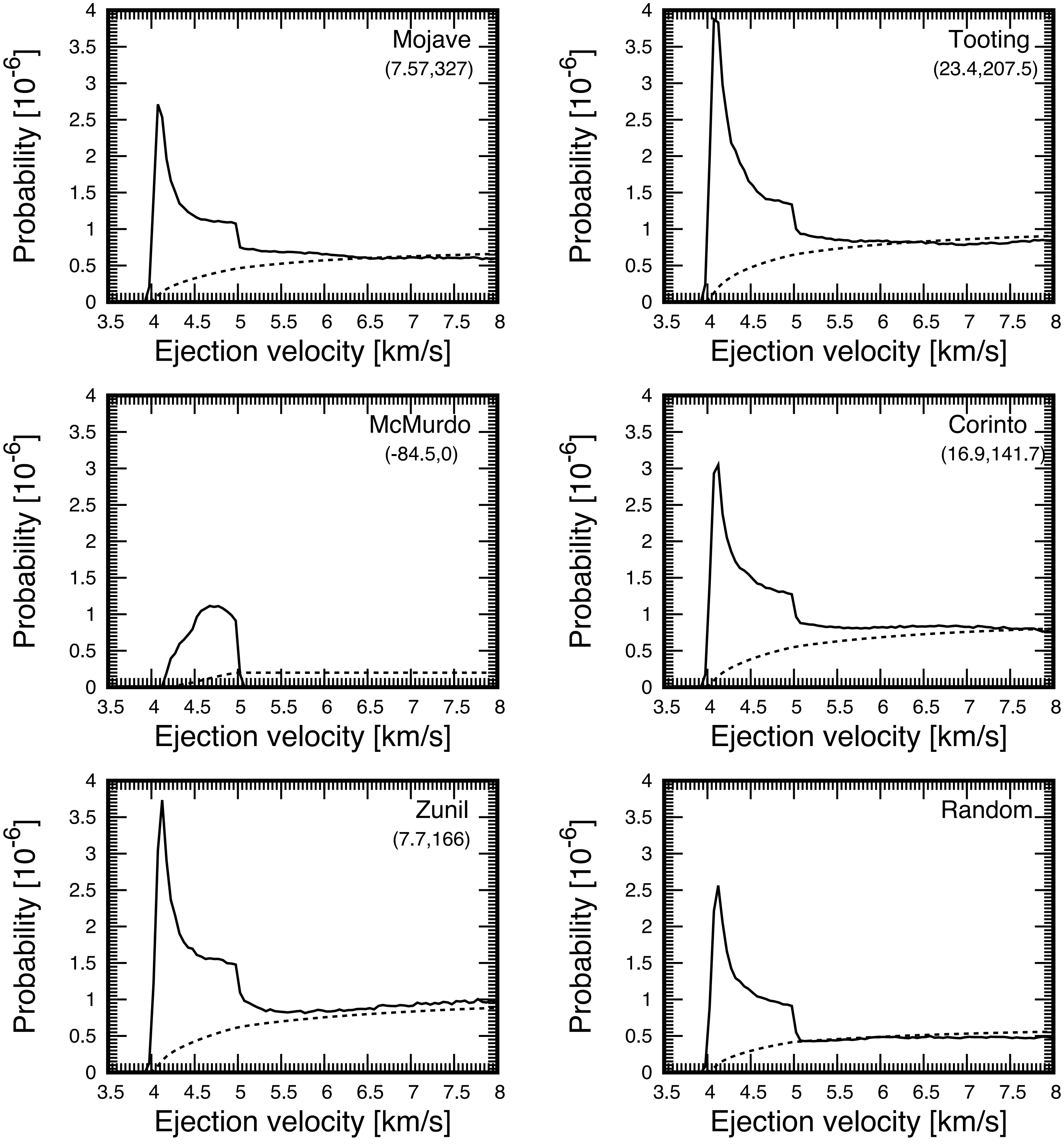

Figure S12. Impact probability for Phobos as a function of ejection velocity from the surface of Mars (solid line). From left to right and from top to bottom panels, the cases of Mojave, Tooting, McMurdo, Corinto, and Zunil, and the fully random case are shown, respectively. The dashed line shows the cumulative impact probability. Latitude and longitude of the craters in degrees are shown in the parentheses in the panels.

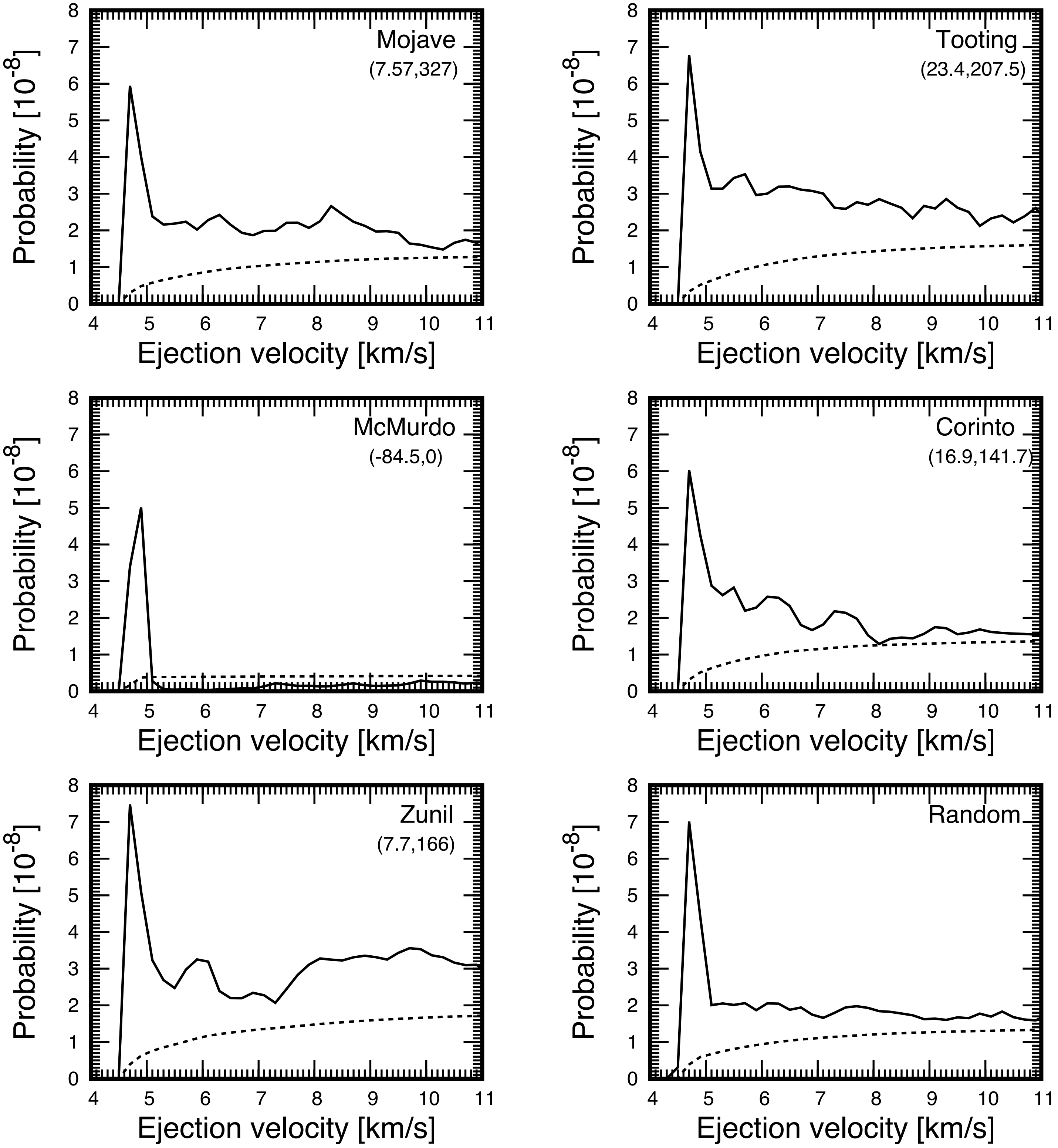

Figure S13. Same as Figure S12, but for the case of Deimos.

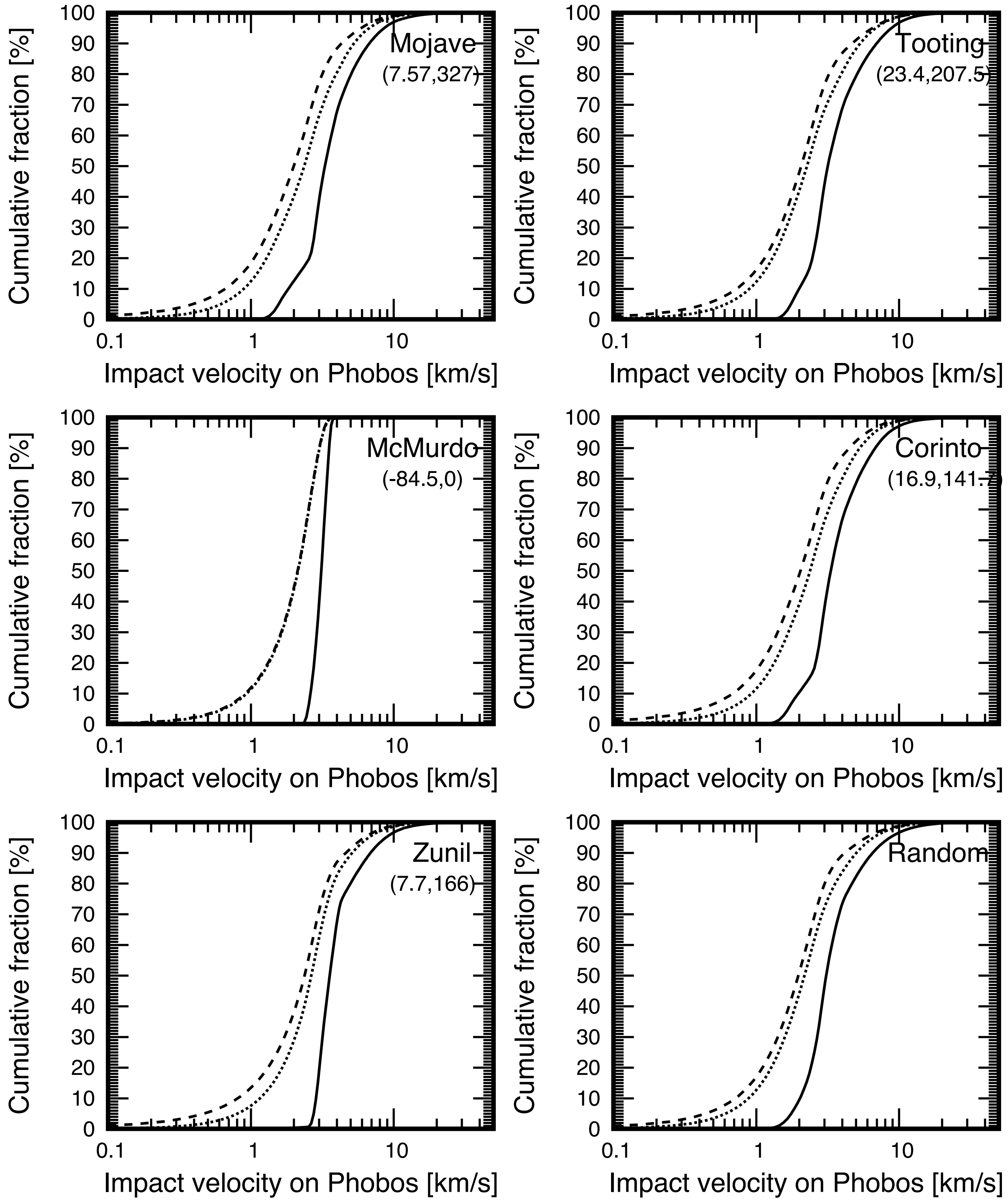

Figure S14. Cumulative distribution of impact velocity to Phobos as a function of impact velocity to Phobos. From left to right and from top to bottom panels, the cases of Mojave, Tooting, McMurdo, Corinto, and Zunil, and the fully random case are shown, respectively. Solid line represents impact velocity. Dashed and dotted lines represent normal and tangential components of the impact velocity, respectively.

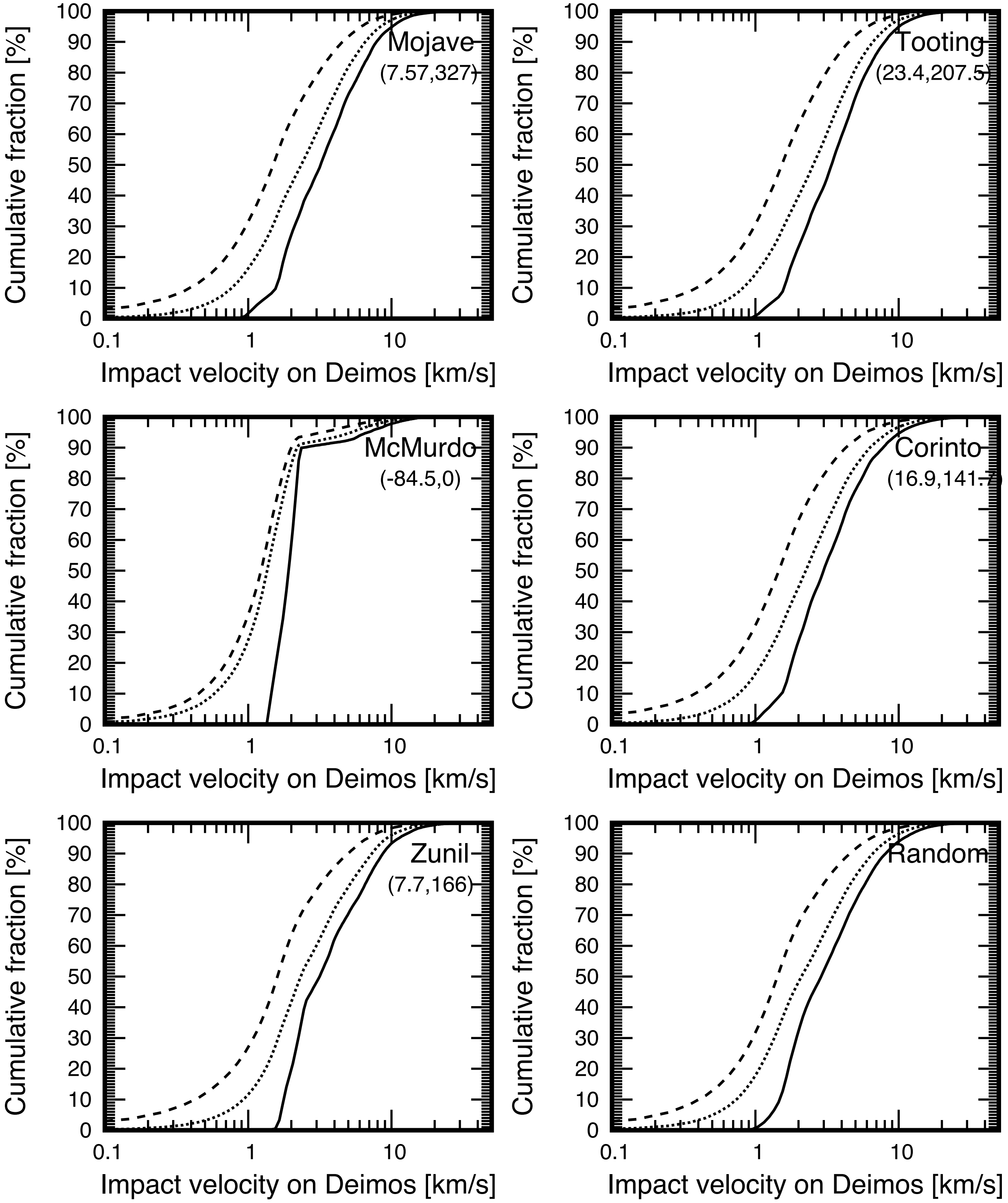

Figure S15. Same as Figure S14, but for the case of Deimos.

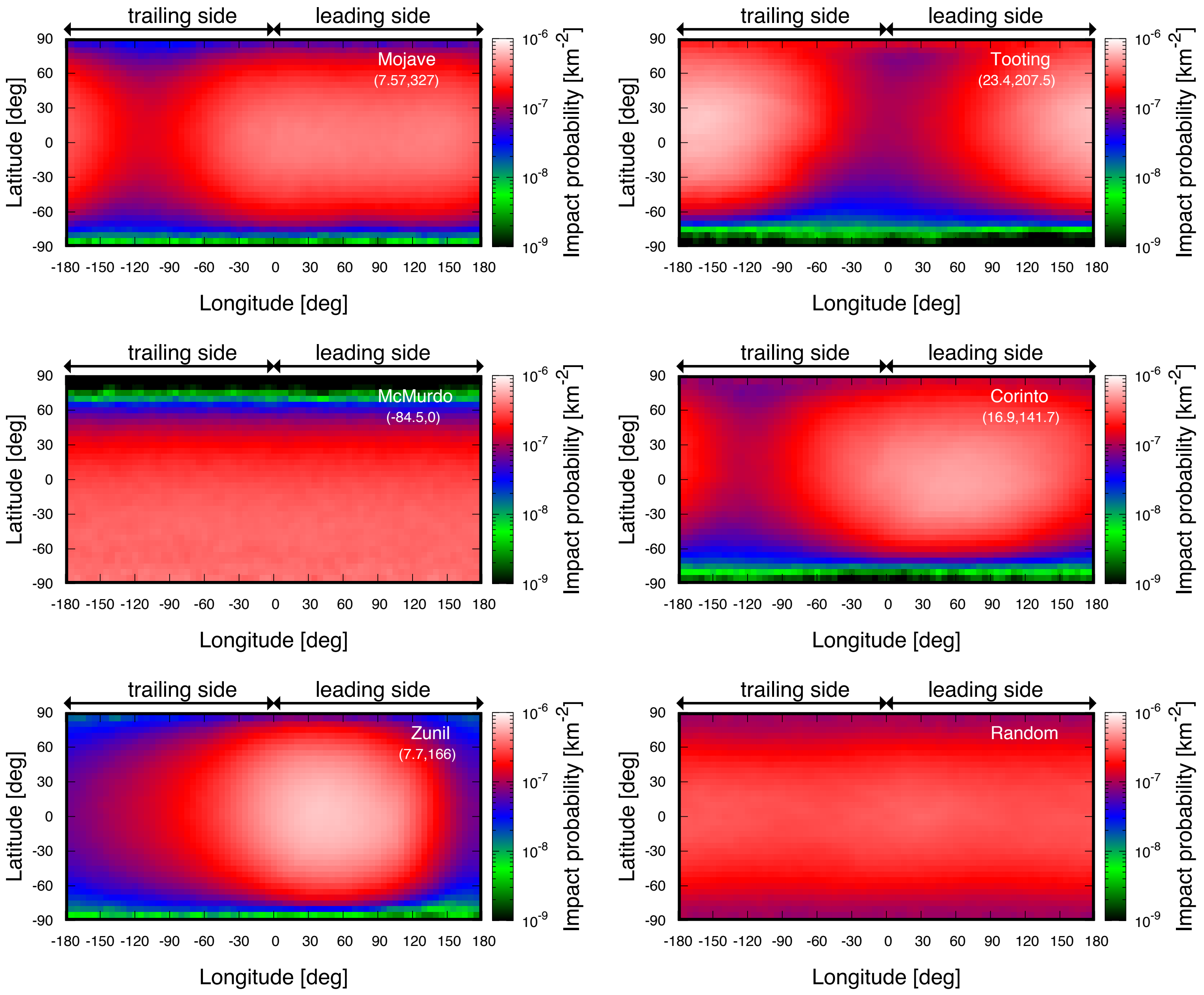

Figure S16. Regional probability of impact on the surface of Phobos considering all the colliding ejecta (color contour) as a function of longitude and latitude of Phobos. Assuming that Phobos is tidally locked, 0 degrees in longitude represents the center of the far side of Phobos to Mars and −180/+180 degrees represents the center of the near side of Phobos to Mars. The longitude of +90 degrees points toward the orbital direction of Phobos and that of −90 degrees points toward the direction opposite to the orbital direction. From left to right and from top to bottom panels, the cases of Mojave, Tooting, McMurdo, Corinto, and Zunil, and the fully random case are shown, respectively. Latitude and longitude of the craters in degrees are shown in the parentheses in the panels.

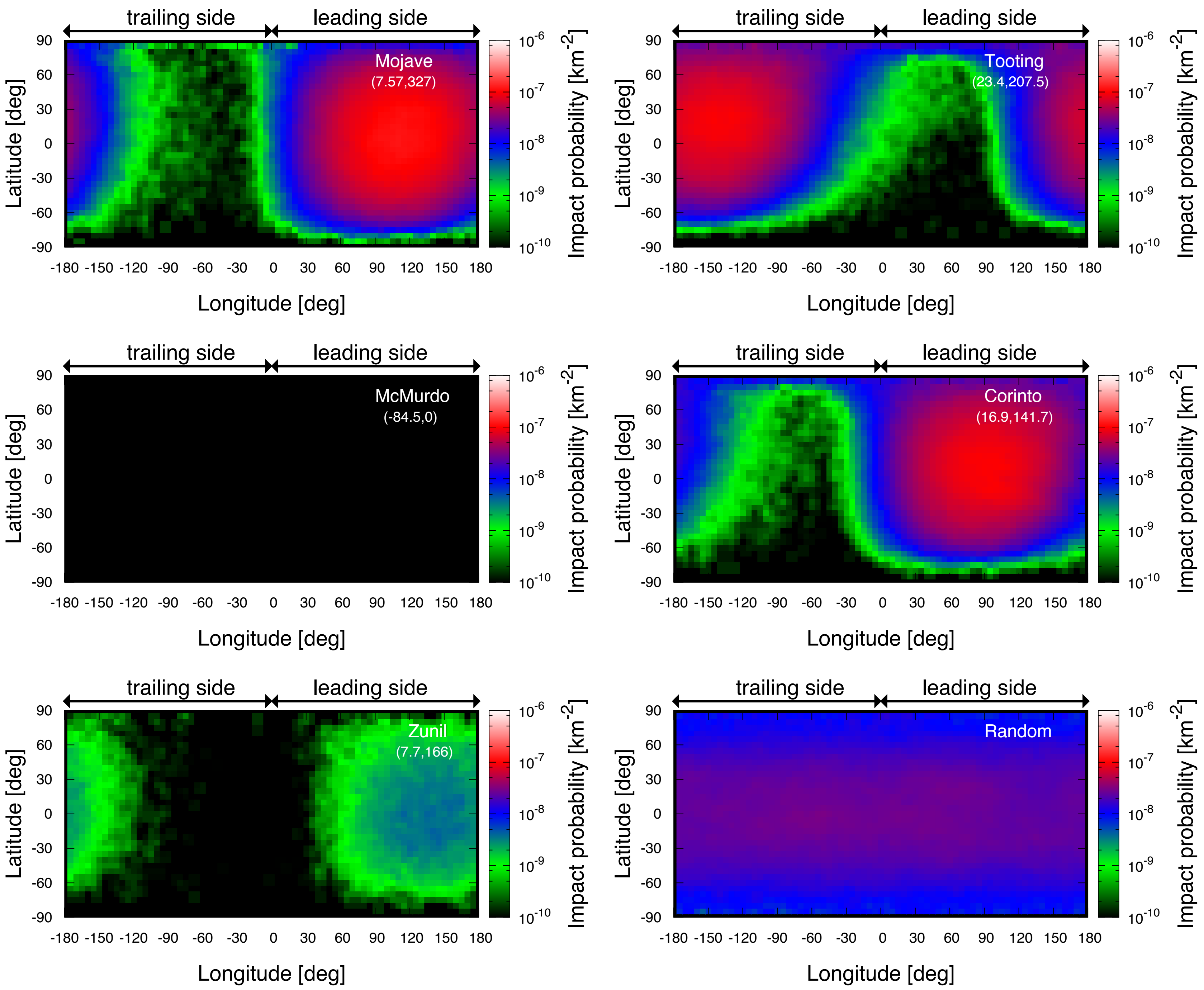

Figure S17. Regional probability of impact on the surface of Phobos for ejecta with impact velocities smaller than 2km/s.

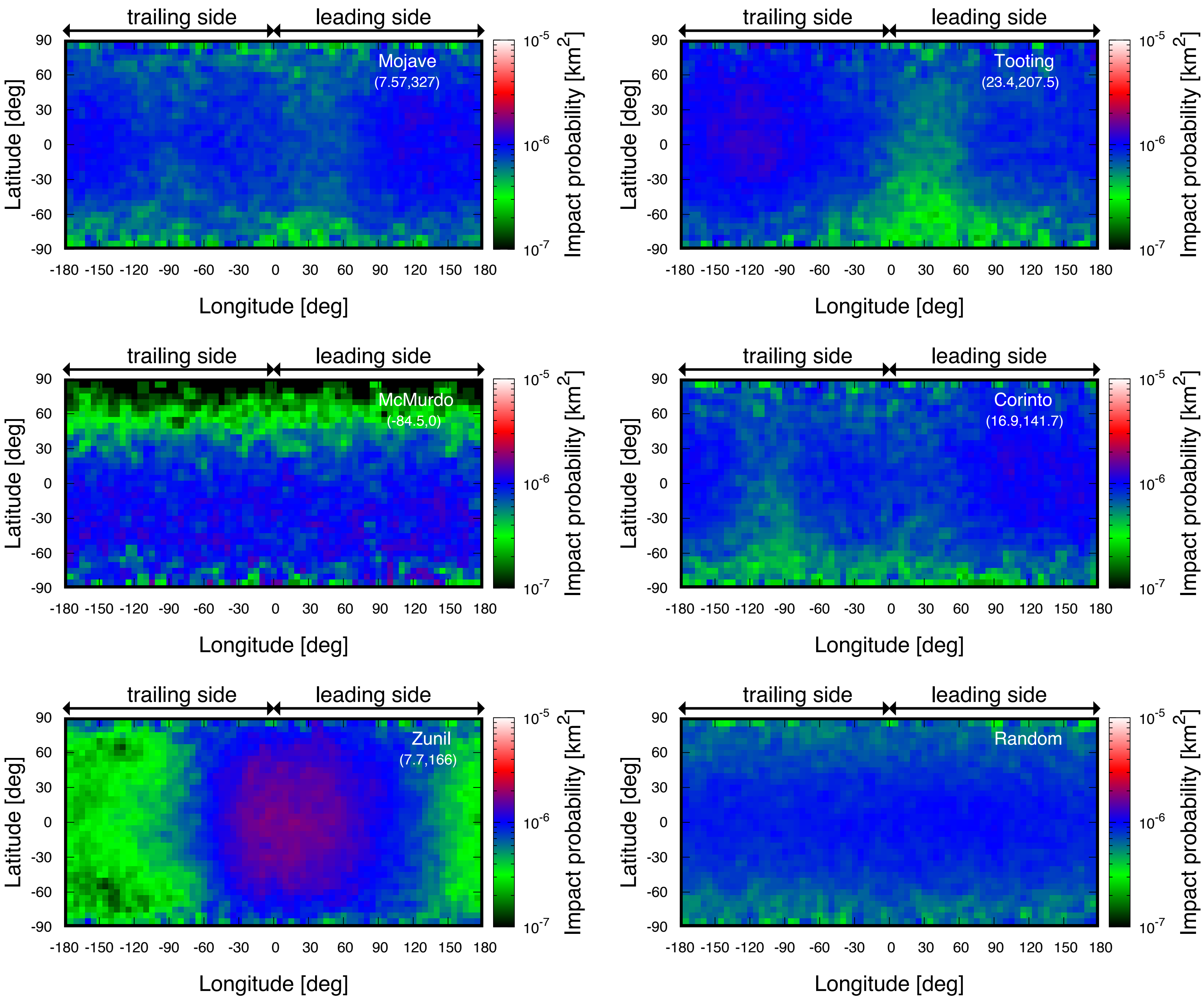

Figure S18. Same as Figure S16, but for the case of Deimos.

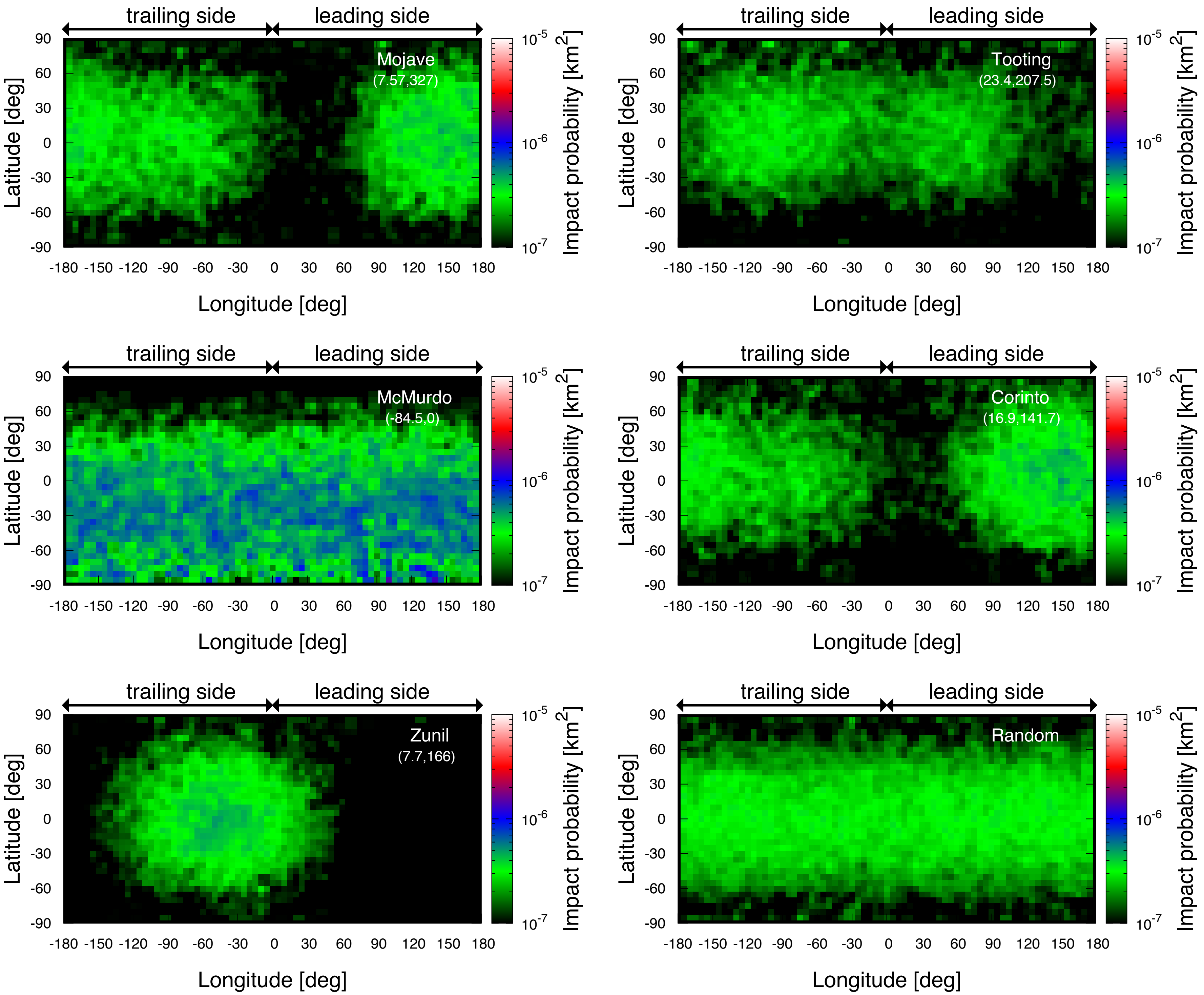

Figure S19. Same as Figure S17, but for the case of Deimos.

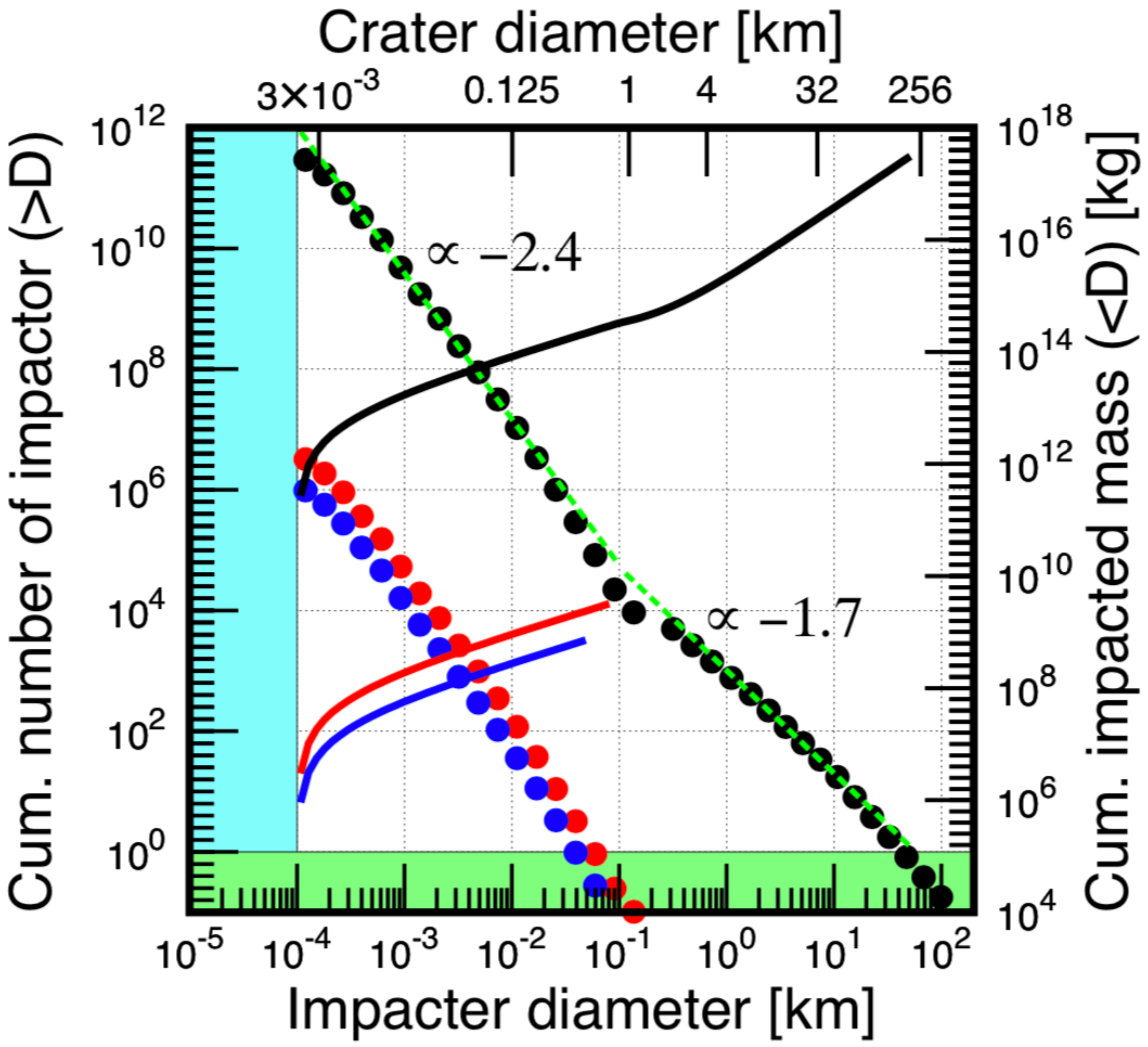

Figure S20. Impactor SFDs and cumulative impactor masses of the natural asteroids to Mars, Phobos and Deimos for the past 500 Myr. The dots are the impactor SFDs of Mars (black), Phobos (red) and Deimos (blue) obtained from crater SFD on Mars, respectively. Dashed green lines represent fitted lines of impactor SFD of Mars for *D*<0.1 km and *D*>0.1 km. Solid lines represent the cumulative impactor masses of natural asteroids to Mars (black), Phobos (red) and Deimos (blue), respectively. Cyan region represents where impactor diameter is smaller than *D*<10^{-4} km. Green region represents where impactor SFD is below one.

Name	Latitude (deg)	East Longitude (deg)	Diameter (km)	Estimated age (Myr)
Mojave	7.57	327.4	58	3–5[a]
Tooting	23.4	207.5	29	2–10[b]
McMurdo	-84.5	0.0	23	2–30[b]
Corinto	16.9	141.7	13.5	2[c]–3.2[d]
Zunil	7.7	166	10.1	0.1–1[b]

Table S1. The locations and diameters of the five fresh craters on Mars considered in this study.

Notes:
[a]Werner et al. (2014) based on a crater chronology model.
[b]Hartmann et al. (2010) based on a crater chronology model.
[c]Hartmann et al. (2010) based on the data by Malin et al. (2006).
[d]Golombek et al. (2014) based on the superposition relationship between the Corinto secondaries and the older-dated lava unit.
